# Tubulin‐binding peptide RR‐171 derived from human umbilical cord serum displays antitumor activity against hepatocellular carcinoma via inducing apoptosis and activating the NF‐kappa B pathway

**DOI:** 10.1111/cpr.13241

**Published:** 2022-05-03

**Authors:** Donglie Zhu, Cheng Fang, Zelong Yang, Yanjie Ren, Fengrui Yang, Shi Zheng, Mingzuo Jiang, Xiangxia Miao, Duoduo Liu, Biliang Chen, Xuebiao Yao, Yong Chen

**Affiliations:** ^1^ Department of Hepatobiliary Surgery, Xijing Hospital Fourth Military Medical University Xi'an China; ^2^ Department of Hand and Foot Surgery The Air Force Hospital of Northern Theater of People's Liberation Army of China Shenyang China; ^3^ Department of Hepatobiliary Surgery, Eastern Hepatobiliary Surgery Hospital Second Military Medical University Shanghai China; ^4^ Department of Gynecology and Obstetrics, Xijing Hospital Fourth Military Medical University China; ^5^ MOE Key Laboratory for Membraneless Organelles & Cellular Dynamics and Hefei National Laboratory for Physical Sciences at the Microscale University of Science and Technology of China Hefei China; ^6^ Department of Gastroenterology and Hepatology, Jinling Hospital Medical School of Nanjing University Nanjing China; ^7^ Department of General Practice Xianyang Central Hospital Xianyang China

## Abstract

**Objectives:**

Hepatocellular carcinoma (HCC) still presents a high incidence of malignant tumours with poor prognosis. There is an urgent need for new therapeutic agents with high specificity, low toxicity and favourable solubility for the clinical treatment of HCC.

**Materials and Methods:**

The bioactivity of human umbilical cord serum was investigated by proteomics biotechnology and a primitive peptide with certain biological activity was identified. The antitumour effect of RR‐171 was detected by cell viability assay in vitro, and determined by subcutaneous xenograft models assay and miniPDX assay in vivo. Pull‐down experiments were conducted to identify the potential targeting proteins of RR‐171. Immunofluorescence assay and tubulin polymerization assay were conducted to explore the relationship between RR‐171 and α‐tubulin. Fluorescence imaging in xenograft models was used to explore the biodistribution of RR‐171 in vivo. A phosphospecific protein microarray was performed to uncover the underlying signalling pathway by which RR‐171 induces tumour cell death.

**Results:**

The results indicated that RR‐171 could be effective in the treatment of HCC in vivo and in vitro. RR‐171 could aggregate significantly in solid tumours and had no obvious systemic toxicity in vivo. RR‐171 could interact with α‐tubulin and activate the NF‐Kappa B pathway in HCC cells.

**Conclusions:**

Taken together, RR‐171 exhibited significant antitumour activity against HCC in vivo and in vitro and could potentially be used in the clinical application of HCC.

## Graphical abstract

RR‐171 derived from human umbilical cord serum, which exhibited significant antitumour activity against HCC in vivo and in vitro. It could not only induce apoptosis and inhibit tubulin polymerization in HCC cells but also activate the NF‐kappa B signalling pathway in HCC cells.

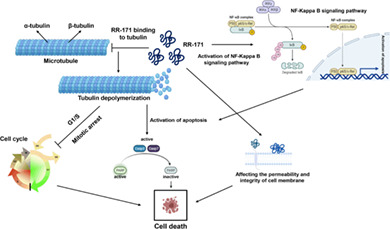

## 1 INTRODUCTION

Hepatocellular carcinoma (HCC) is the dominant type of primary liver cancer, and the prognosis of HCC is poor.^1^ Although a number of risk factors such as HBV, HCV, alcohol, nonalcoholic fatty liver disease, diabetes, dietary factors, etc, may be responsible for the high incidence of HCC, HBV and HCV are known to be the most important aetiological factors for HCC.^2^ HCC can be treated with multidisciplinary therapy, such as surgical resection, radiofrequency ablation, systemic chemotherapy and liver transplantation (LT), while surgical resection and LT are considered to be effective approaches among these options.^3^, ^4^ Sorafenib is currently considered to be a first‐line treatment for advanced HCC patients, but sorafenib causes many side effects, including palmar‐plantar erythrodysesthesia, diarrhoea, weight loss, and hypertension, and patients still have a poor prognosis.^5^, ^6^ There is an urgent need for new agents with better curative effects and fewer side effects in clinical application.

Peptides are involved in chemistry and biology, and they play an important regulatory role in biological growth, metabolism, immunity and disease. Peptides have shown excellent prospects in the field of clinical application, especially for cancer therapy, due to a variety of biological functions.^7^ Peptides possess strong plasticity and good tissue permeability with low toxicity, and they still show remarkable pharmacokinetic characteristics.^8^

Peptides could be applied in cancer therapy, serving multiple roles, such as direct anticancer drugs targeting drug carriers, endocrine hormones and tumour vaccines.^9^, ^10^, ^11^ Anticancer peptides are the general name of a series of peptides with antitumour activity.^12^ Many anticancer peptides are derived from natural plants or animals, and others are screened from phage peptide libraries and chemosynthetic peptide libraries.^13^, ^14^

Umbilical cord blood, which is an important material guarantee for foetal survival, has complex components, including a variety of proteins and lipids. Umbilical cord serum has abundant multiple cytokines, which play an outstanding role in cell proliferation, differentiation and immune regulation.^15^ However, research on the relationship between umbilical cord serum and cancer has scarcely been found to date. A previous study reported that natural killer (NK) cells from umbilical cord blood could be used in combination with bevacizumab as a novel therapeutic target for colorectal cancer.^16^ Mesenchymal stem cells derived from the human umbilical cord were designed to deliver, replicate and assemble into new adenoviruses against HCC.^17^ Therefore, anticancer components could be found in umbilical cord blood, which may inspire the discovery of new agents for clinical treatment. In this study, we explored the effect and mechanism of the novel peptide RR‐171 derived from human umbilical cord serum, which exhibited noteworthy antitumour activity against hepatocellular carcinoma.

## 2 MATERIALS AND METHODS

### 2.1 Umbilical cord blood extraction

Serum samples were provided by Xijing Hospital (Xi'an, Shaanxi, China), and this study was approved by the ethics committee of Xijing Hospital. We selected 5 healthy pregnant women from Xijing Hospital who were pregnant for the first time and had a natural vaginal delivery. None of these subjects had complications or had a history of smoking or alcohol abuse. None of them had taken medications within three months or were infected with infectious diseases. After the delivery of the foetus, we rapidly extracted umbilical cord blood. One hour later, the samples were centrifuged at 1000 *g* for 30 min at room temperature, and we collected the upper layer of clear serum. Finally, the samples were stored under refrigeration at −130°C.

### 2.2 Reversed‐phase liquid chromatography and mass spectrometry analysis

Reversed‐phase liquid chromatography analysis and mass spectrometry analysis had been performed previously.^18^

### 2.3 Peptide modification

Based on the original peptide derived from umbilical cord serum, RR‐171 was synthesized by adding four arginines to the amino terminus and carboxyl terminus of the original peptide to increase the solubility of the original peptide. The control peptide RR‐170 was obtained by replacing valine and leucine with lysine according to RR‐171. The peptides were synthesized using standard solid‐phase synthetic peptide chemistry and purified using HPLC. An Agilent‐6125B (SQ LC/MSD, CA, USA) mass spectrometer was used to analyse peptides. HPLC was performed to separate the peptides using a GS‐120‐5‐C18‐BIO chromatographic column (4.6 × 250 mm), and the column temperature was set at 35 °C. The mobile phase consisted of 0.1% trifluoroacetic acid in 100% acetonitrile (solvent A) and 0.1% trifluoroacetic acid in 100% water (solvent B). The flow rate was 1 ml/min, and the wavelength was 220 nm. Mass spectrometry analysis of peptides and HPLC chromatograms of the purified peptides are provided in Figure S1. The online software INNOVAGEN (https://pepcalc.com/) was used to predict the physicochemical properties of peptides.

### 2.4 Cell culture and reagents

A normal human liver cell line (QSG‐7701) and liver cancer cells (Hep3B, MHCC97H, HepG2 and HCCLM6) were purchased from American Type Culture Collection (ATCC). The other cells were a gift from Dr. Qu and Dr. Chen. A normal human liver cell line (QSG‐7701) was cultured in RPMI‐1640 medium (HyClone), and cancer cell lines were cultured in Dulbecco's Modified Eagle Medium (DMEM) (HyClone) containing 10% foetal bovine serum (Biological Industries). Moreover, these cells were cultured in an incubator at 37°C with 5% CO_2_.

Necrostatin‐1, PD150606 and Z‐VAD‐FMK were obtained from Sigma Aldrich. The microtubule (MT)‐stabilizing agents epothilone B and paclitaxel and the MT‐destabilizing agents fosbretabulin and nocodazole were purchased from MedchemExpress Inc. Each of the reagents was dissolved in DMSO (Sigma Aldrich) and diluted to the appropriate concentrations.

### 2.5 Circular dichroism (CD) spectroscopy analysis

CD spectra were collected with a JASCO J‐500A spectropolarimeter at room temperature (190–260 nm wavelength range, 1 mm path length, 1.0 nm band width and 100 nm/min scanning speed). The synthetic peptides were obtained from ChinaPeptides (China), and the solutions of peptides were prepared in 1 mg/ml phosphate buffer saline (PBS) (HyClone) solutions. The samples of peptides were filtered through centrifugal filters to prepare for CD measurements. The spectra of each sample were accumulated, and the data were averaged. The CDNN program was used to predict the secondary structures of peptides.

### 2.6 Cell viability assay

Cell viability was measured by a Cell Counting Kit‐8 kit (Sigma). Cells were seeded at 3 × 10^3^ cells per well in 96‐well plates for 24 h, and various concentrations of peptide solutions were added to the wells for another 48 h. Then, the cells were incubated with CCK‐8 reagents for 2 h at 37°C, and a microplate reader (Thermo, USA) was used to measure the absorbance at 450 nm. IC_50_ values were calculated according to nonlinear regression analysis using GraphPad Prism software. Necrostatin‐1, PD150606 and Z‐VAD‐FMK were dissolved in DMSO and added to the wells for 12 h, after which peptides (100 μg/ml) were added to the wells for another 48 h. Finally, OD_450_ values were measured as mentioned above.

### 2.7 Flow cytometry

#### 2.7.1 Flow cytometry analysis for the detection of FITC‐positive cells

HCC cells were cultured in 12‐well plates and incubated in incubators at 37°C overnight. Then, peptides labelled with FITC were added to the wells, after which cells were digested at different time points and prepared into a single‐cell suspension. Flow cytometry was used to analyse HCC cell suspensions treated at different times (0, 0.1, 1, 6, 12, 24 and 48 h), and the proportion of FITC‐positive cells was counted to calculate the penetration rate of peptides into HCC cells.

#### 2.7.2 Flow cytometry analysis for cell cycle

HCC cells were cultured in 6‐well (3 × 10^5^ cells per well) plates in incubators at 37°C overnight, after which cells were treated with peptides (100 μg/ml) for another 48 h. Then, the cells were digested and fixed in 70% ethanol for 24 h and stained with 50 μg/ml propidium iodide (Sigma Aldrich) for 30 min in the dark. Finally, cell cycle analysis was performed using a flow cytometer (BD, Biosciences).

#### 2.7.3 Flow cytometry analysis for cell apoptosis

HCC cells were cultured at 3 × 10^5^ cells per well in 6‐well plates, and then peptides (100 μg/ml) were added to the wells for another 24 h. Then, the cells were digested and stained with Annexin V‐FITC (Sigma Aldrich) and propidium iodide (Sigma Aldrich) reagents at room temperature for 20 min in the dark. A flow cytometer (BD, Biosciences) was used to detect the fluorescence of Annexin V‐FITC and propidium iodide. The rate of apoptotic and necrotic cells was calculated using DIVA software (BD, Biosciences).

### 2.8 Western blotting analysis

Following the different treatments with peptides, RIPA buffer (Thermo Fisher Scientific, USA) with proteinase and phosphatase inhibitors (Roche) was used to extract total proteins from cells. The total proteins were quantified by a BCA protein quantification kit (Thermo Fisher Scientific, USA). Subsequently, samples were separated on a 10%–12% sodium dodecyl sulfate‐polyacrylamide gel electrophosesis (SDS‐PAGE) gel and transferred to polyvinylidene fluoride (PVDF) membranes (Millipore, USA). PBS containing 5% nonfat milk was used to block the membranes at room temperature for 1 h. Membranes were then incubated with primary antibodies overnight at 4°C. On the second day, the membranes were washed with tris‐buffered saline with Tween (TBST) buffer (washed three times, 5 min per wash) and incubated with secondary antibodies for 1 h at room temperature. Finally, enhanced chemiluminescence reagent (Thermo Fisher Scientific, USA) was used to process the membranes, after which the membranes were exposed to chemiluminescence imaging systems (Bio–Rad).

The following antibodies were used: monoclonal antibody cyclinD1 (CST, 1:1000), monoclonal antibody cyclinE1 (CST, 1:1000), monoclonal antibody p21 (CST, 1:1000), monoclonal antibody phospho‐Rb (CST, 1:1000), monoclonal antibody p53 (CST, 1:1000), monoclonal antibody phospho‐p53 (CST, 1:1000), monoclonal antibody caspase 3 (CST, 1:1000), monoclonal antibody cleaved‐caspase 3 (CST, 1:1000), monoclonal antibody caspase 7 (CST, 1:1000), monoclonal antibody cleaved‐caspase 7 (CST, 1:1000), monoclonal antibody PARP (CST, 1:1000), monoclonal antibody cleaved‐PARP (CST, 1:1000), monoclonal antibody BLNK (CST, 1:1000), monoclonal antibody phospho‐BLNK (CST, 1:1000), monoclonal antibody IKKβ (CST, 1:1000), phospho‐IKKβ (CST, 1:1000), monoclonal antibody IκBα (CST, 1:1000), phospho‐IκBα (CST, 1:1000), monoclonal antibody p65 (CST, 1:1000), monoclonal antibody phospho‐p65 (CST, 1:1000), monoclonal antibody α‐tubulin (CST, 1:1000) and monoclonal antibody actin (CST, 1:1000).

### 2.9 LDH release assay

The level of LDH in the cell culture supernatant was detected by an LDH assay kit (Thermo Fisher Scientific, USA). LDH released from damaged HCC cells treated with peptides was measured to estimate the integrity of the cell membrane. Maximum LDH release was generated from cells treated with 0.9% Triton X‐100 as a positive control. The collected samples reacted with the substrate mix to perform an enzymatic reaction in a 96‐well enzymatic assay plate. A microplate reader (Thermo Fisher Scientific, USA) was employed to detect the absorption values at 450 nm. The percentage of LDH release was calculated as 100 × (experimental LDH release‐spontaneous LDH release)/(maximum LDH release − spontaneous LDH release).

### 2.10 Scanning electron microscopy (SEM)

Morphological examination was performed with SEM. HCC cells were cultured at 2 × 10^5^ cells per well in 6‐well plates, after which peptides (100 μg/ml) were added into the wells for another 48 h. The samples were fixed with 3% glutaraldehyde for more than 24 h and washed twice with distilled water. The samples were dehydrated using an acetonitrile gradient and dried under vacuum. Next, samples were mounted on aluminium stubs, after which the samples were sputter‐coated with gold using a sputter coater (Agar Scientific, UK) to prevent beam charging effects. SEM analysis was performed using a Quanta 450 FEG SEM (FEI, USA).

### 2.11 Immunofluorescence microscopy

HCC cells were cultured in coverslips and incubated with FITC‐labelled peptides (100 μg/ml) for different time periods (0, 0.1, 1, 6, 12, 24 and 48 h) at 37°C. Then, coverslips were fixed in 4% paraformaldehyde for 20 min at room temperature and permeabilized with 0.1% Triton X‐100 for 15 min followed by washing with PBS. Then, blocking buffer (containing 2% bovine serum albumin) was added to the slides, and the slides were incubated for 2 h at room temperature followed by staining with DAPI (Sigma, Aldrich) to visualize nuclear DNA.

Determining the colocalization relationship between RR‐171 and α‐tubulin was basically consistent with the previous experimental process. HCC cells were cultured in coverslips and incubated with FITC‐labelled peptides at different concentrations at 37°C. After permeabilization, samples were incubated with primary antibody against α‐tubulin (Abcam) followed by incubation with a secondary antibody. DNA was counterstained with DAPI as described above.

To observe microtubule aggregation during the interphase and mitotic phases, cells were pretreated with different peptides or agents (RR‐170, RR‐171, RR‐171 + Epothilone B, Fosbretabulin, Nocodazole, Paclitaxel and Epothilone B) at appropriate concentrations. Tubulin was labelled with green fluorescence, actin was labelled with red fluorescence and DNA was counterstained with DAPI labelling blue fluorescence. Cells were imaged using a scanning confocal microscope (LSM880 Carl Zeiss).

### 2.12 Subcutaneous xenograft models

In this study, thirty BALB/c nude mice (Beijing Vital River Animal Centre) (6–8 weeks) were randomly divided into three groups (negative control, RR‐170 and RR‐171 groups), with 10 rats in each group maintained under specific pathogen‐free (SPF) conditions. HCC cells were seeded into dishes to 70%–80% confluence, resuspended and diluted to a final concentration of 5 × 10^6^/ml. We injected 200 μl of cell suspension into the flanks of nude mice by subcutaneous injection. Dead subjects were eliminated during the tumorigenesis process. When the tumour size reached 100 mm^3^, peptide solutions were injected intravenously, and PBS was injected as a control. Body weight, tumour size and animal condition were monitored every 4 days. The mice were sacrificed 24 days postinjection, and the major organs and subcutaneous tumour masses were removed and subjected to HE staining. After euthanasia, serum samples of nude mice were collected. The haematology parameters in serum were detected by an automatic biochemical analyser (Olympus, Japan), including ALB (albumin), ALP (alkaline phosphatase), ALT (alanine aminotransferase), AST (aspartate aminotransferase) and TBIL (total bilirubin).

### 2.13 Fluorescence imaging in xenograft models

BALB/c nude mice were purchased from Beijing Vital River Animal Centre (6–8 weeks). HCC cells (5 × 10^6^) resuspended in 150 μl PBS solutions were injected subcutaneously into the flanks of nude mice. When the tumour size reached 100 mm^3^, tumour‐bearing mice were randomly divided into three groups (with 10 rats in each group) and injected with Cy7 (MedChemExpress, Inc.), Cy7‐RR‐170 and Cy7‐RR‐171 intravenously. Then, anaesthetized mice were imaged using in vivo imaging system (IVIS) spectrum (PerkinElmer, Inc.) at 1, 3, 6, 12, 24 and 48 h. After 6 h injection, three mice in each group were anaesthetized and sacrificed, and tumours were collected for imaging. Twenty‐four hours after injection, three mice in each group were anaesthetized and sacrificed, and their major organs were collected for imaging.

### 2.14 HE staining assay

In the above animal experiments, formalin‐fixed major organs and subcutaneous tumour masses were embedded in paraffin blocks and cut into 6‐μm sections, which were adhered to slides. After being dried, paraffin sections were dewaxed with xylene (5 min × 5 times) and hydrated with gradient ethanol (5 min). Then, the slices were stained with haematoxylin (Sigma, Aldrich) for 10 min and washed off. Then, colour separation with 1% hydrochloric acid was performed for 10 s, followed by bluing and counterstaining in eosin (Sigma, Aldrich) for 30 s. After HE staining, tumour necrosis and tissue structures of kidney, liver, and lung were observed under a light microscope.

### 2.15 MiniPDX assay

Liver cancer specimens were obtained from biopsy samples collected during surgical treatment and confirmed by pathology at Xijing Hospital. Tumour sample acquisition was approved by the ethics committees of Xijing Hospital and agreed upon by each patient via written informed consent. BALB/c nude mice (Beijing Vital River Animal Centre) (4–6 weeks) were used for subcutaneous implantation. Hanks' balanced salt solution was used to wash the tumour samples three times, and the fatty, connective and necrotic tissues were removed. Then, the samples were minced, and collagenase was used to digest the samples at 37°C. Next, samples were transferred to Hanks' balanced salt solution (HBSS)‐washed capsules. The capsules containing tumour cells were implanted subcutaneously, and each mouse received 3 capsules. The peptide solutions were injected into the mice through the tail vein for seven days, after which the capsules were removed, and the tumour/control (T/C) rate was calculated. The T/C rate was calculated as follows: (Mean relative light units [RLU] of the treatment group on Day 7‐Mean RLU on Day 0)/(Mean RLU of the vehicle group on Day 7‐Mean RLU on Day 0).

### 2.16 Pull down assay

First, the resin container was gently reversed, streptavidin magnetic beads were mixed into a homogenate and 80 μl of homogenate was placed into a marked centrifuge tube. Then, the centrifuge tube was put into the magnetic rack followed by magnetic separation for 1 min, and the supernatant was discarded. Next, we added 1 ml PBS into the centrifuge tube and mixed it upside down, followed by magnetic separation for 1 min. The control group was mixed with blank magnetic beads and streptavidin magnetic beads, and the experimental group was mixed with 1 mg biotin‐labelled peptide and streptavidin magnetic beads. Two milligrams of total protein were added to both groups and incubated overnight at 4°C. After magnetic separation for 1 min, the samples were washed with PBST three times, and the supernatant was discarded. Then, 80 μl RIPA buffer and 20 μl 6 × loading buffer were added to the centrifuge tube, which was boiled for 5–10 min. The supernatant was collected after centrifugation at 16,000 *g* for 5 min. The obtained supernatants were separated by SDS‐PAGE electrophoresis, and silver staining was used to detect whether there were different bands between the groups. The protein samples of the groups were identified by mass spectrometry, and the differential proteins were analysed.

### 2.17 Tubulin polymerization assay

The in vitro tubulin polymerization assay was performed with a tubulin polymerization assay kit (Cytoskeleton, USA) based on the kit's protocol. Hep3B cells were treated with RR‐170, RR‐171, RR‐171+epothilone B, fosbretabulin, nocodazole, paclitaxel and epothilone B. In brief, purified porcine tubulin was diluted in G‐PEM buffer containing 0.1 mM MES, 1 mM EGTA, 0.5 mM MgCl_2_, 4 mM glycerin and 1 mM GTP. Then, the tubulin solution and the peptides were transferred into a 96‐well plate at the designed concentration. Epothilone B and paclitaxel were used as microtubule stabilizing controls, and fosbretabulin and nocodazole were used as microtubule destabilizing controls. Tubulin polymerization was measured by a fluorescence microplate reader at 37°C (FLUOstar Omega, Germany).

### 2.18 Phosphor‐specific protein microarray analysis

A phosphor‐specific protein microarray was purchased from Full Moon Microsystems Inc. The flow chart for phosphor‐specific protein microarray analysis is provided in Figure S2. The antibody microarray was composed of 304 antibodies, among which 157 antibodies were phosphoproteins and 144 were their unphosphorylated counterparts. Analysis of the microarray was performed by Wayen Biotechnologies (Shanghai, China) following the manufacturer's protocol. The phosphorylation ratio of each protein was calculated according to the following formula: phosphorylation ratio = phosphorylated value/unphosphorylated value.

### 2.19 Statistical analysis

Statistical analysis was performed by SPSS 22.0 (SPSS, Chicago, IL). A paired Student's *t*‐test was used to compare two groups. Comparisons among three groups were analysed using one‐way ANOVA followed by Bonferroni's post‐hoc test. We considered *p* < 0.05 to be statistically significant.

## 3 RESULTS

### 3.1 Identification and modification of the antitumour peptides and the measurement of their secondary structure and physicochemical properties

**FIGURE 1:**
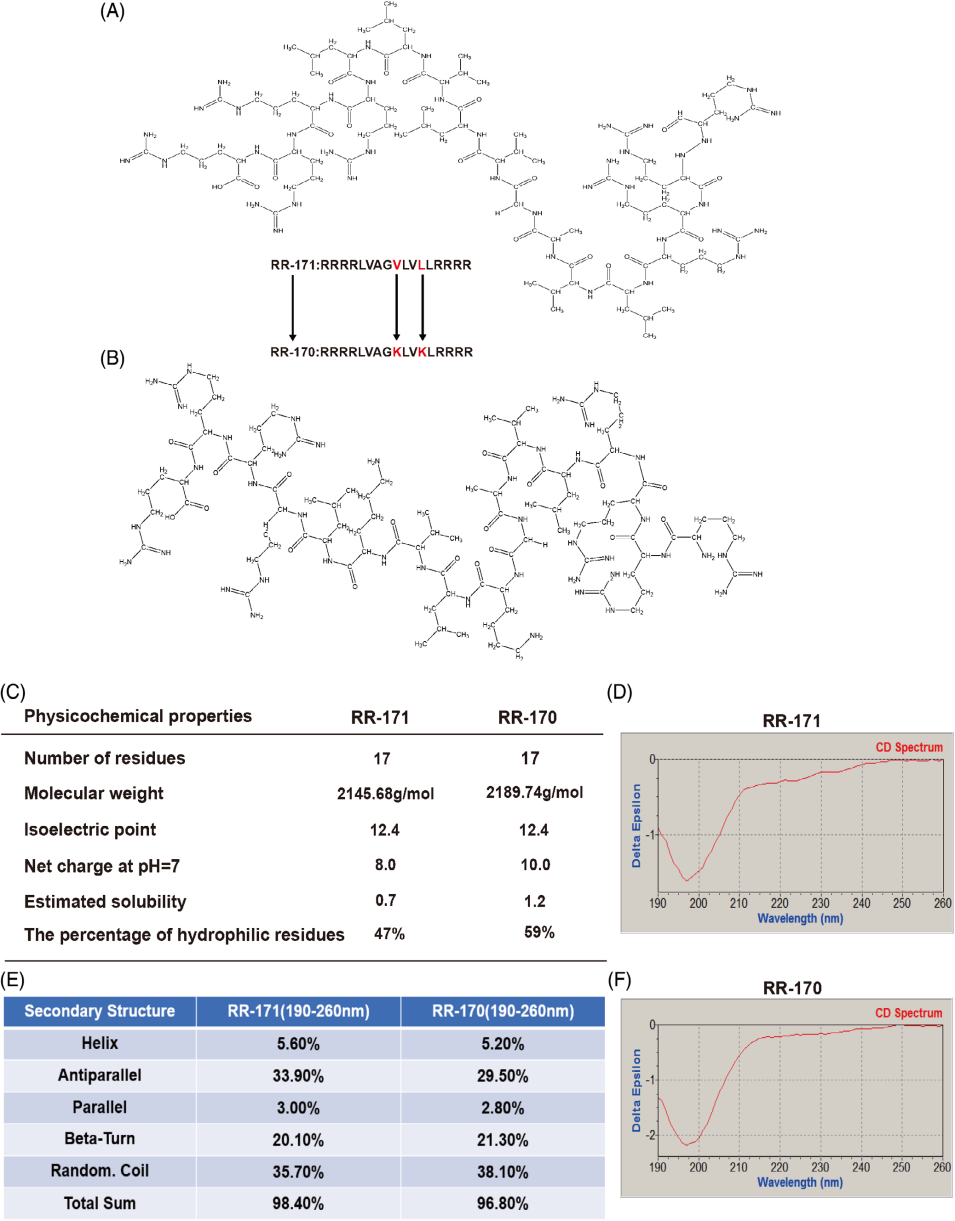
Identification and modification of the antitumour peptides and the measurement of their secondary structure and physicochemical properties. (A,B) The chemical structural formulas of RR‐171 and RR‐170 and the composition of their amino acid sequences. (C) The physicochemical properties of peptides, including the number of residues, molecular weight, isoelectric point, net charge, estimated solubility and percentage of hydrophilic residues. (D) The circular dichroism spectrum of RR‐171. (E) The prediction of the secondary structure of peptides, including helix, antiparallel, parallel, beta‐turn and random coil. (F) The circular dichroism spectrum of RR‐170

In this project, we used reverse‐phase high‐performance liquid chromatography‐mass spectrometry (HPLC/MS) to analyse the low‐abundance proteins in umbilical cord serum and found a component that inhibited cell viability. Through further mass spectrometry analysis, we obtained the original peptide. However, the solubility of the original peptide was very poor, and we modified this peptide to enhance the solubility and finally obtained the modified peptide RR‐171 based on the original peptide (Figure 1A). To further exclude nonspecific factor effects, such as concentration and acid base, a control peptide based on RR‐171 was synthesized. The control peptide RR‐170 was obtained by replacing valine and leucine with lysine (Figure 1B). We used the online software INNOVAGEN to predict the physicochemical properties of peptides. Circular dichroism (CD) spectra were used to analyse the secondary structural features of the peptides. The results showed that the molecular weight of RR‐171 was 2145.68 g/mol, the isoelectric point was 12.4 and the net charge was 8. While the molecular weight of RR‐170 was 2189.74 g/mol, the isoelectric point was 12.4, and the net charge was 10. In the prediction of secondary structure of RR‐171, random coil accounted for 35.7%, antiparallel structure accounted for 33.9% and beta‐turn accounted for 20.1%. The proportion of random coils in RR‐170 was 38.1%, antiparallel structure was 29.5% and beta‐turns was 21.3% (Figure 1C–F).

### 3.2 RR‐171 exhibited an inhibitory effect on the growth of various tumour cells in vitro, especially liver cancer cells

**FIGURE 2:**
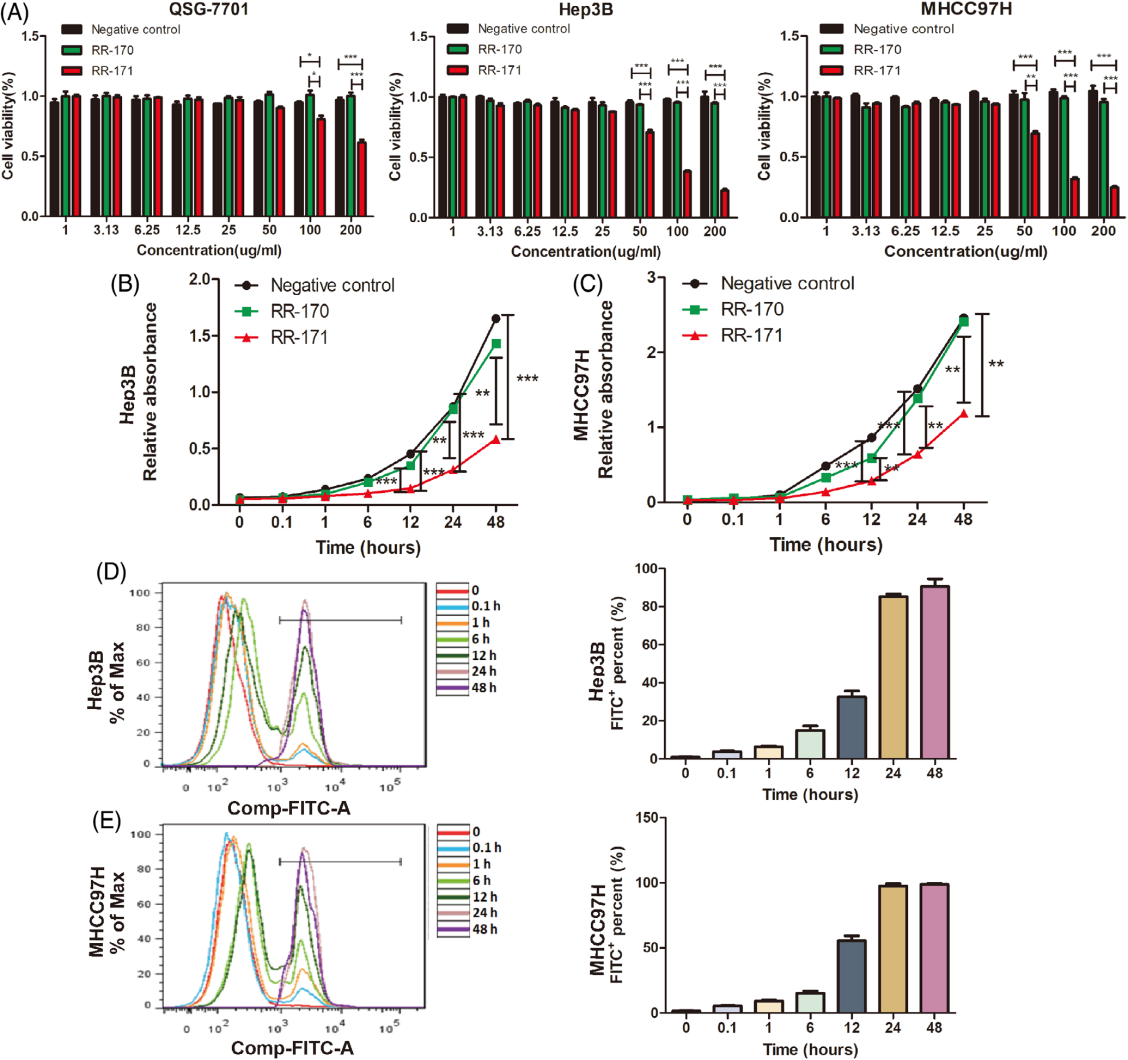
RR‐171 exhibited an inhibitory effect on the growth of various tumour cells in vitro, especially liver cancer cells. (A) QSG‐7701, Hep3B and MHCC97H cell lines were incubated with RR‐170 or RR‐171 at different concentrations. The cell viability was measured by CCK8 assay. (B,C) After treatment with RR‐170 or RR‐171, the proliferation rate of HCC cell lines was tested by CCK‐8 assay at different time points (0, 0.1, 1, 6, 12, 24 and 48 h). (D,E) The percentage of FITC‐positive cells was detected by flow cytometry assay in HCC cell lines treated with RR‐171 at different time points (0, 0.1, 1, 6, 12, 24 and 48 h). The data are presented as the mean ± standard error of the mean (SEM). *p < 0.05, **p < 0.01

RR‐171 was first evaluated for the growth inhibition test by the CCK‐8 assay. The results confirmed that RR‐171 was cytotoxic to various human tumour cells in vitro, including liver cancer, lung cancer, pancreatic cancer, ovarian cancer, colon cancer, breast cancer and gastric cancer. The IC_50_ values of RR‐171 are presented in Table 1. We found that RR‐171 also inhibited the growth of normal fibroblasts and human normal liver cell lines. However, the toxicity of RR‐171 to normal fibroblasts and human normal liver cell lines was much lower than the toxicity of RR‐171 to tumour cells. As shown in Table 1, liver cancer cell lines were more sensitive to RR‐171 than other tumour cells. Therefore, we chose liver cancer cell lines as the research object of RR‐171. After treatment with different concentrations of RR‐171, the viability of the liver cancer cell lines decreased with increasing RR‐171 concentration. No obvious changes were observed in the RR‐170 group (Figure 2A). The results of detection of cell viability of other cancer cell lines treated with RR‐171 or RR‐170 are shown in Figure S3.

**TABLE 1:** Evaluation for the growth inhibition test of RR‐171 and the relevant values are presented as follow

Cell lines	IC50 (μg/ml)	LogIC50	95%CI (IC50)	95%CI (LogIC50)
MHCC97h	80.07	1.903	61.9–103.6	1.792–2.015
Hep3B	81.53	1.911	65.23–101.9	1.814–2.008
HepG2	22.92	1.360	16.94–31.01	1.229–1.492
Huh7	66.53	1.823	56.03–79.00	1.748–1.898
HCCLM3	78.02	1.892	66.10–92.09	1.820–1.964
HCCLM6	80.46	1.906	73.03–88.64	1.864–1.948
7701	287.9	2.459	263.4–314.7	2.421–2.498
PANC‐1	98.95	1.995	78.78–124.3	1.896–2.094
A549	74.92	1.875	61.36–91.48	1.788–1.961
SW480	105.7	2.024	92.76–120.4	1.967–2.080
A2780	76.73	1.885	61.79–95.27	1.791–1.979
SKBR‐3	123.7	2.092	108.9–140.6	2.037–2.148
MKN45	90.75	1.958	75.23–109.5	1.876–2.039
FBPS	173.2	2.239	132.5–226.5	2.122–2.355

*Note*: IC50 was calculated by nonlinear regression analysis using GraphPad Prism software.

Abbreviation: 95% CI, 95% confidence interval.

To investigate whether the toxicity of RR‐171 has time‐dependent characteristics, we tested the proliferation rate of Hep3B and MHCC97H cell lines by the CCK‐8 assay. Compared with the negative control (NC) group and RR‐170 group, RR‐171 exhibited an inhibitory effect on tumour cells after 12 h, and the inhibitory effect reached a peak at 48 h (Figure 2B,C). Simultaneously, to verify the proportion of RR‐171 entering tumour cells at different time points, we carried out flow cytometry detection. The percentage of fluorescein isothiocyanate (FITC)‐positive cells clearly increased at 12 h and reached a peak at 48 h in the living cells (Figure 2D,E).

This evidence clearly indicated that RR‐171 was cytotoxic to various tumour cells in vitro, especially to liver cancer cells. RR‐171 exhibited an inhibitory effect on tumour cells after 12 h, and the inhibitory effect reached a peak at 48 h.

### 3.3 RR‐171 inhibited G1/S phase transition and induced apoptosis in HCC cells

**FIGURE 3:**
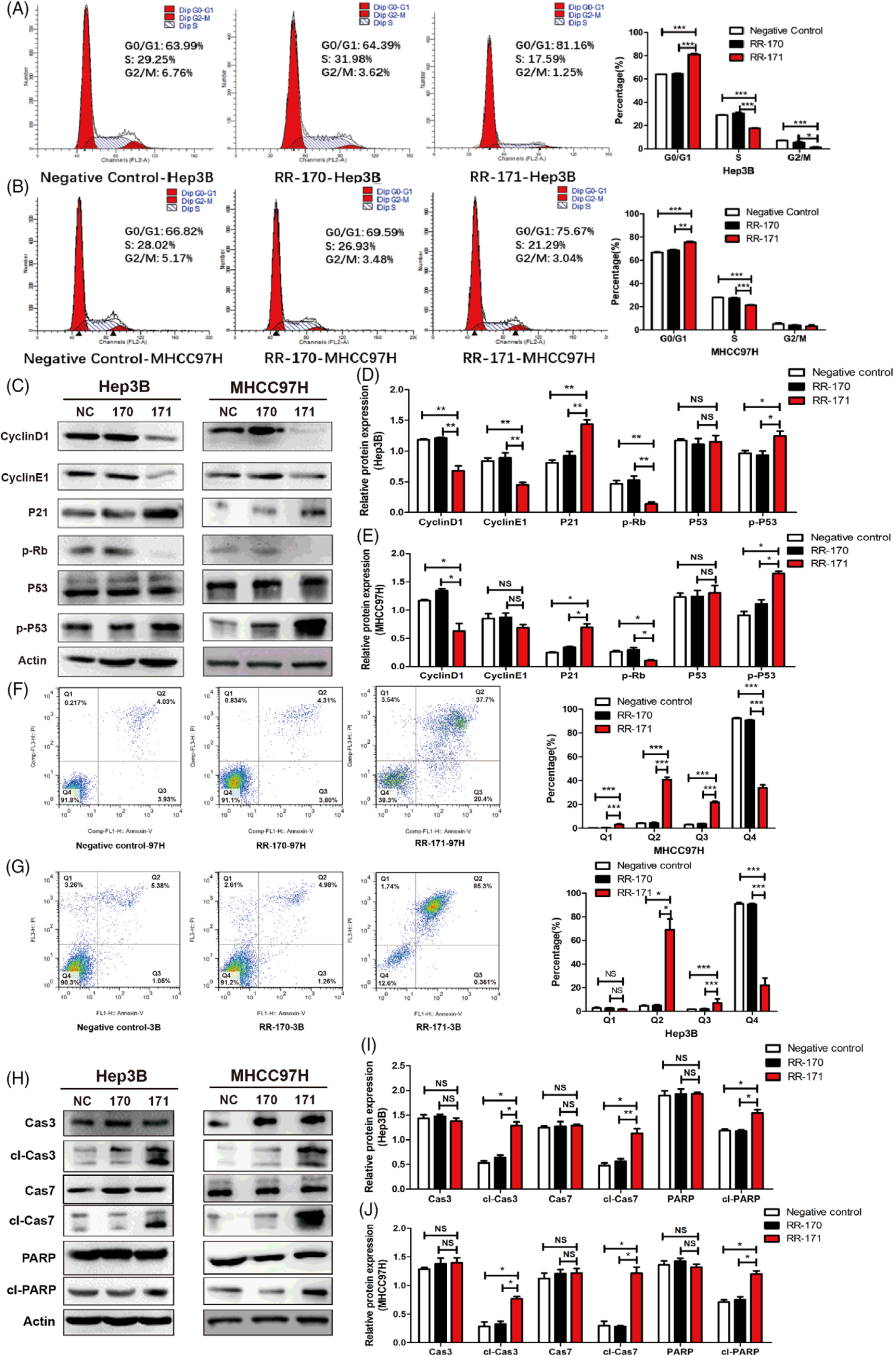
RR‐171 inhibited the G1/S phase transition and induced apoptosis in HCC cells. (A,B) Cell cycle distribution was measured in HCC cell lines treated with RR‐170 or RR‐171 by flow cytometry assay. (C) Western blotting analysis of proteins (cyclinD1, cyclinE1, P21, p‐Rb, P53 and p‐P53) related to the cell cycle in HCC cells treated with RR‐170 or RR‐171. (D,E) Quantification of C from the Western blot experiments. (F,G) Flow cytometry analysis of cell apoptosis in HCC cell lines treated with RR‐170 or RR‐171. (H) Western blotting analysis of proteins (caspase 3, cleaved caspase 3, caspase 7, cleaved caspase 7, PARP and cleaved PARP) related to cell apoptosis in HCC cell lines treated with RR‐170 or RR‐171. (I,J) Quantification of H from the Western blotting experiments. The data are presented as the mean ± standard error of the mean (SEM). *p < 0.05, **p < 0.01, ***p < 0.001

To evaluate whether RR‐171 plays a pivotal role in regulating the cell cycle progression of HCC cells, Hep3B and MHCC97H cells treated with RR‐171 were measured by flow cytometry. The data obtained clearly indicated that the percentage of cells in the G0/G1 phase increased while the percentage of cells in the S and G2/M phases decreased dramatically compared with the NC group and RR‐170 group (Figure 3A,B). Because of these results, we subsequently detected the protein expression level of some key factors that might regulate cell cycle progression. As shown in Figure 3C–E, the levels of CyclinD1, CyclinE1 and p‐Rb were clearly decreased in HCC cells treated with RR‐171 compared with the NC group and RR‐170 group, while the levels of P21 and p‐P53 were conversely increased in the RR‐171 group. These results confirmed that RR‐171 inhibited the G1/S phase transition of the cell cycle in HCC cells.

Given that RR‐171 inhibited the growth of HCC cell lines by inhibiting the G1/S phase transition, we wondered whether RR‐171 affected HCC cell viability via apoptosis induction. To investigate the effect of RR‐171 on cell apoptosis, we implemented flow cytometry analysis in Hep3B and MHCC97H cell lines treated with RR‐171. As shown in Figure 3F,G, we found that RR‐171 treatment increased the percentage of early apoptotic and late apoptotic HCC cells compared with the negative control and RR‐170 groups. Furthermore, we observed that cleaved caspase 3, cleaved caspase 7 and cleaved poly‐ADP ribose polymerase (PARP) were activated in the RR‐171 group. Western blotting assays were conducted to detect the relative protein expression, and cleaved caspase 3, cleaved caspase 7 and cleaved PARP were upregulated in the RR‐171 group compared with the NC group and RR‐170 group. There was no significant change in caspase 3, caspase 7 or PARP protein levels in HCC cells among these groups. Overall, RR‐171 induced apoptosis and activated apoptosis‐related factors in HCC cells.

### 3.4 RR‐171 induced cell death through apoptosis and obviously affected the permeability and integrity of the cell membrane

**FIGURE 4:**
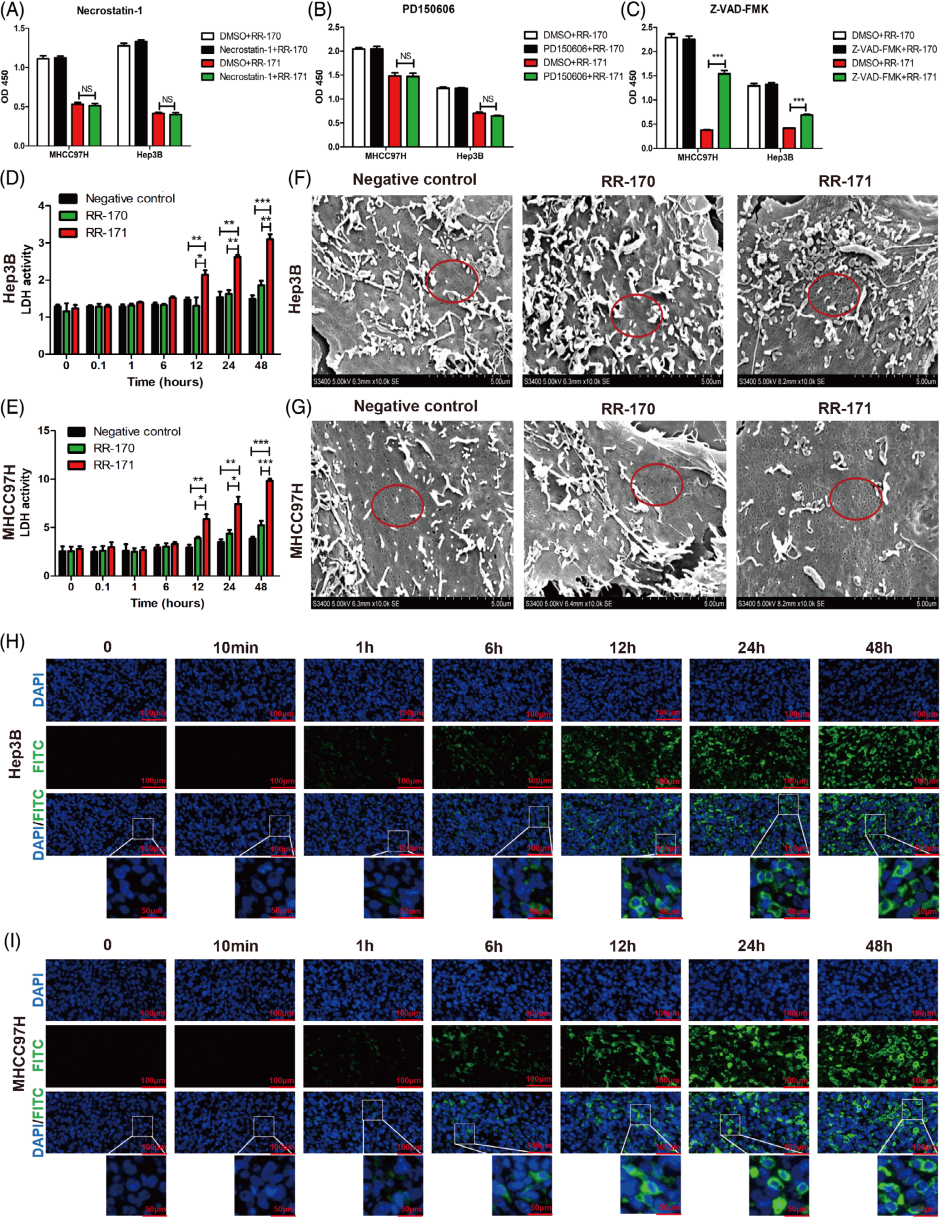
RR‐171 induced cell death through apoptosis and obviously affected the permeability and integrity of the cell membrane. (A–C) CCK‐8 assay showing the OD_450_ of HCC cell lines treated with necrostatin‐1, PD150606 or Z‐VAD‐FMK in four groups (DMSO + RR‐170 group, inhibitors + RR‐170 group, DMSO + RR‐171 group and inhibitors + RR‐171 group). (D,E) LDH release assay was used to detect LDH activity in HCC cells treated with RR‐170 or RR‐171 at different time points (0, 0.1, 1, 6, 12, 24 and 48 h). (F,G) The integrity of the cell membrane was observed by scanning electron microscopy after treatment with RR‐170 or RR‐171. (H,I) Cellular immunofluorescence technology was used to detect RR‐171 activity in HCC cells at different time points (0, 0.1, 1, 6, 12, 24 and 48). Scale bars: top, 100 μm; bottom, 50 μm. The data are presented as the mean ± SEM. **p < 0.01, ***p < 0.001

To further determine the specific death mechanism caused by RR‐171, we used the necrosis inhibitor necrostatin‐1, oncosis inhibitor PD150606 and apoptosis inhibitor Z‐VAD‐FMK to coculture HCC cells with RR‐171 treatment. As shown in Figure 4A–C, the experimental groups were divided into four groups: dimethyl sulfoxide (DMSO) + RR‐170, inhibitors + RR‐170, DMSO + RR‐171 and inhibitors + RR‐171. The data indicated that the apoptosis inhibitor Z‐VAD‐FMK could reverse quite a few cell deaths caused by RR‐171 treatment; however, no significant effect was observed on necrostatin‐1 and PD150606. These results further proved that RR‐171 induced cell death through apoptosis.

The level of LDH in the cell culture supernatant will increase when the integrity of the cell membrane is destroyed. To clarify whether RR‐171 affects the permeability of the cell membrane, we conducted a lactate dehydrogenase (LDH) release assay to detect LDH activity in HCC cells after treatment with RR‐171 or RR‐170. In the present study, the LDH level was higher in the RR‐171 group than in the negative control and RR‐170 groups, and LDH activity reached a peak at 48 h in HCC cells (Figure 4D,E). To visualize the process by which RR‐171 acts on cells at different time points, we used cellular immunofluorescence technology for detection. Consistent with the aforementioned results (Figure 2D,E), the bioactivity of RR‐171 appeared to be obviously detected at 12 h and reached a peak at 48 h (Figure 4H,I).

To confirm our ideas, we used scanning electron microscopy (SEM) to observe the integrity of the cell membrane from the perspective of morphology. As shown in Figure 4F,G, we found that there were many microvilli and micropores on the cellular surface. The micropores on the cellular surface increased, and the integrity of the cell membrane was destroyed significantly in the RR‐171 group compared with the NC group and RR‐170 group in HCC cells. These results provided conclusive evidence that RR‐171 affected the permeability and integrity of the cell membrane in HCC cells.

### 3.5 RR‐171 could aggregate significantly in solid tumours in vivo and inhibit the growth of subcutaneous xenograft tumours of HCC in nude mice

**FIGURE 5:**
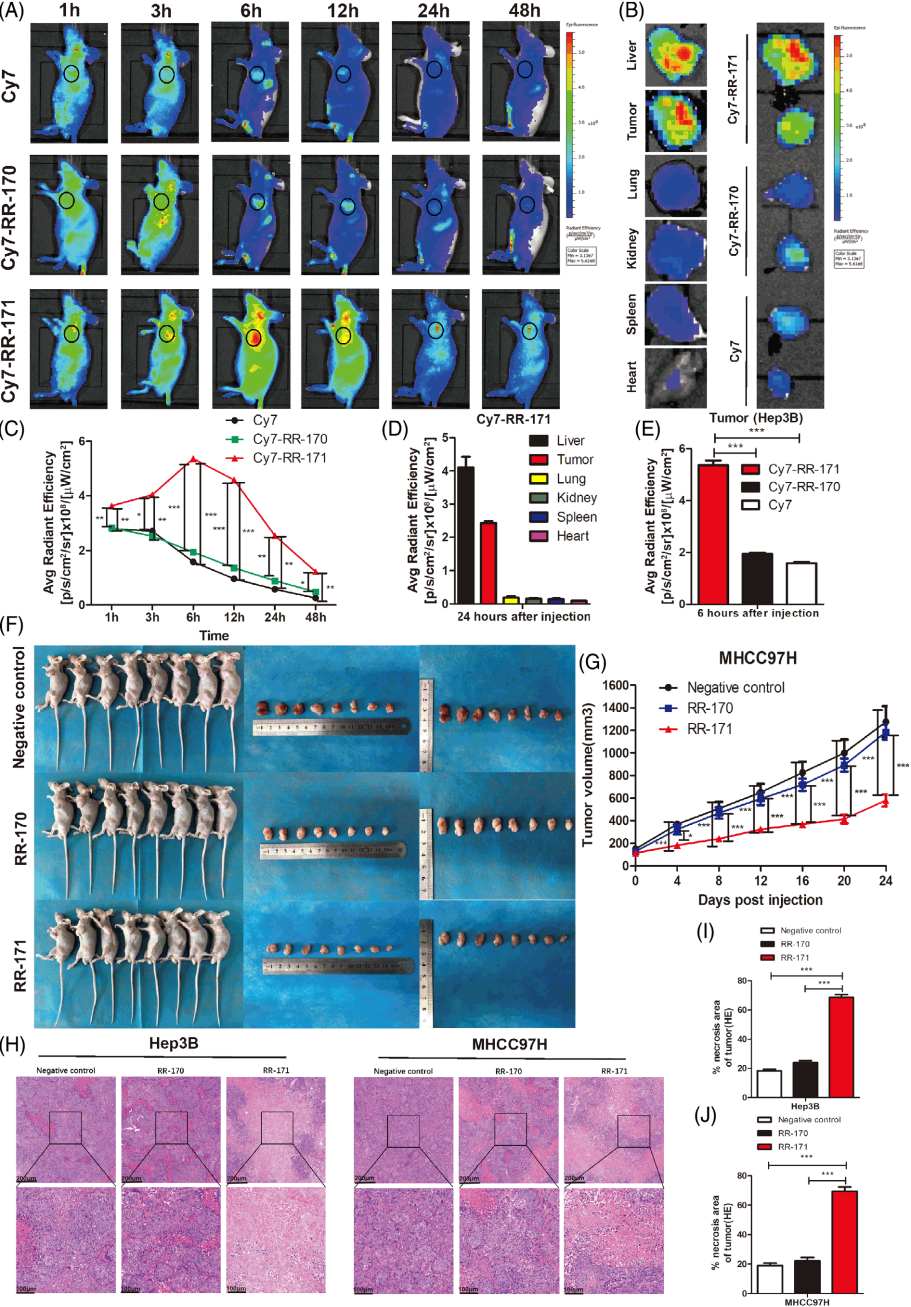
RR‐171 was capable of significantly accumulating in solid tumours and inhibited the growth of subcutaneous xenograft tumours in nude mice. (A) Fluorescein Cy7 and peptides labelled with fluorescein Cy7 were injected into nude mice. The fluorescence images of tumour‐bearing mice were observed by IVIS Spectroscopy at different time points (1, 3, 6, 12, 24 and 48 h) including three groups (Cy7, Cy7‐RR‐170, and Cy7‐RR‐171 group). (B) Fluorescence images of the organs and transplanted tumours from nude mice. (C) Analysis of the average radiant efficiency of A. (D) Analysis of the average radiant efficiency of the major organs at 48 h after injection. (E) Analysis of the average radiant efficiency of the transplanted tumours from different groups (Cy7, Cy7‐RR‐170, and Cy7‐RR‐171 group) at 6 h after injection. (F) Images of xenograft tumour‐bearing nude mice and transplanted tumours. (G) The measurement of tumour volumes from the negative control, RR‐170 and RR‐171 groups. (H) HE staining images of necrotic areas of tumours from the negative control, RR‐170 and RR‐171 groups. Scale bars: top, 200 μm; bottom, 100 μm. (I,J) Quantification of H from the HE staining experiments. The data are presented as the mean ± SEM. *p < 0.05, **p < 0.01, ***p < 0.001

In addition to exploring the biological activity of RR‐171 in vitro, we have also conducted extensive research on the biological effects of RR‐171 in vivo. First, we explored the tumour‐targeting ability and biodistribution of RR‐171 in vivo by labelling it fluorescently with Cy7. When the subcutaneous xenograft tumour of HCC grew to the appropriate size in nude mice, we injected fluorescein Cy7 and peptides labelled with fluorescein Cy7 into nude mice through the tail vein. We recorded the fluorescence images of mice by in vivo imaging system (IVIS) spectra at different timepoints (1, 3, 6, 12, 24, 48 h). The accumulation of Cy7‐RR‐171 in the tumour was much higher than the accumulation of Cy7 and Cy7‐RR‐170. Cy7‐RR‐171 reached the maximum degree of enrichment at 6 h after injection (Figure 5A,C). Then, the intensity of the fluorescence signal gradually weakened over time as a result of the gradual metabolism of the drugs in vivo. We removed the major organs and transplanted tumours from nude mice 24 h after injection. We explored the biodistribution of Cy7‐RR‐171 in vivo by detecting the fluorescence signal intensity of these organs and transplanted tumours. As shown in Figure 5B and D, the fluorescence intensity of liver and tumour tissue was the strongest, while the fluorescence intensity of other organs (including lung, kidney, spleen and heart) was very weak. Moreover, we removed the transplanted tumours of the groups (Cy7, Cy7‐RR‐170, and Cy7‐RR‐171 group) and detected the fluorescence signal intensity at 6 h after injection. As shown in Figure 5B,E, we found that the MFI (mean fluorescence intensity) of Cy7‐RR‐171 in the tumour was much higher than the MFI in the Cy7 and Cy7‐RR‐170 groups by quantitative analysis, which was in agreement with the above results (Figure 5C). These data suggest that RR‐171 could aggregate significantly in solid tumours in vivo.

To examine the antitumour activity of RR‐171 in vivo, we injected HCC cell suspensions into the flanks of nude mice by subcutaneous injection to establish subcutaneously transplanted tumour models. When the subcutaneous xenograft tumour grew to the appropriate size in nude mice, we injected peptide‐containing PBS solutions into the mice intravenously. As shown in Figure 5F,G, we removed all the subcutaneous tumours 24 days postinjection and measured the volume of these tumour masses. We found that RR‐171 significantly inhibited the growth of subcutaneous xenograft tumours in nude mice compared with the negative control and RR‐170 groups. Subsequently, HE staining was performed on the tumour samples from the above three groups, and necrotic areas of the tumour were analysed statistically (Figure 5H). The data indicated that necrotic areas of tumours in the RR‐171 group were significantly larger than the necrotic areas of tumours in the negative control and RR‐170 groups (Figure 5I,J). This evidence proved that RR‐171 significantly inhibited the growth of subcutaneous xenograft tumours of HCC in vivo.

### 3.6 RR‐171 had no obvious systemic toxicity in vivo or the results of the MiniPDX assay

**FIGURE 6:**
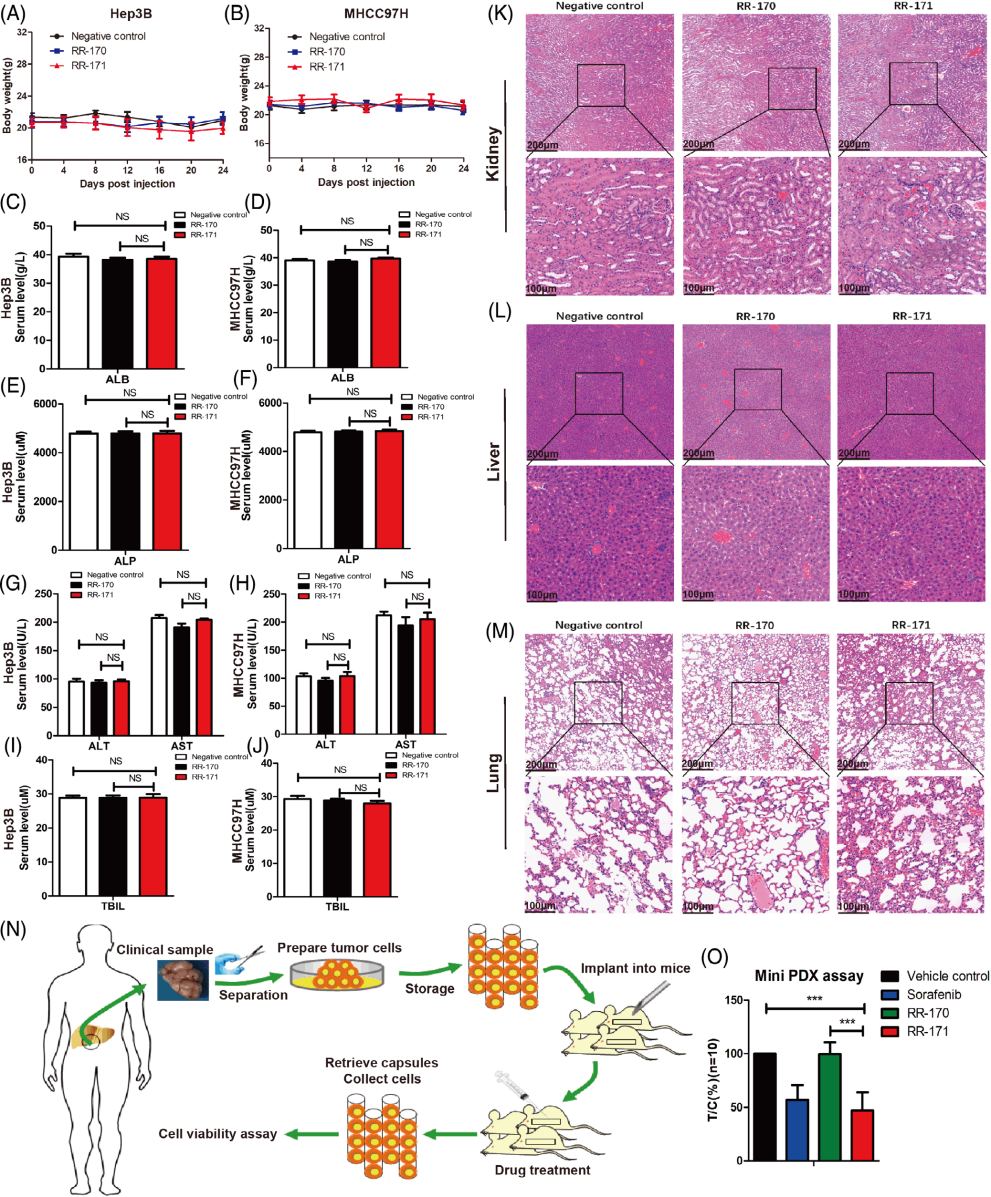
RR‐171 had no obvious systemic toxicity in vivo, and the results of the miniPDX assay. (A,B) The measurement of body weight from different groups during 24 days of injection treatment. (C–J) The serum levels of ALB (albumin), ALP (alkaline phosphatase), ALT (alanine aminotransferase), AST (aspartate aminotransferase) and TBIL (total bilirubin) were detected in xenograft tumour‐bearing nude mice of different groups. (K–M) HE staining images of major organs from tumour‐bearing nude mice after the injection of different peptides. Scale bars: top, 200 μm; bottom, 100 μm. (N) The general schema diagram of mini‐PDX models. (O) The tumour/control (T/C) rate of each group of miniPDX models. The data are presented as the mean ± SEM. ***p < 0.001

To assess the systemic toxicity of RR‐171 in vivo, we first measured the body weight of nude mice during the 24 days of treatment. We found that there was no obvious weight loss in the RR‐171 group (Figure 6A,B). Then, we evaluated the hepatotoxicity of RR‐171 by detecting the levels of ALB (albumin), ALP (alkaline phosphatase), ALT (alanine aminotransferase), AST (aspartate aminotransferase) and TBIL (total bilirubin) in serum samples. The values of these indices in serum samples were all within the normal range (Figure 6C–J), indicating that there was no evident hepatotoxicity of RR‐171, although RR‐171 accumulated significantly in the liver. Subsequently, to assess organ toxicity due to RR‐171 administration, we performed HE staining on the kidney, liver and lung tissues from tumour‐bearing mice and observed pathological slices by light microscopy. As shown in Figure 6K–M, no significant histopathological abnormalities were found in the RR‐171 group compared with the negative control and RR‐170 groups. Overall, this evidence indicated that RR‐171 had no obvious systemic toxicity in vivo.

To further demonstrate the results obtained in the xenograft nude mouse models, a minipatient‐derived xenograft (PDX) assay was conducted to evaluate the antitumour efficacy. The flow chart of the integral process of the miniPDX assay is shown in Figure 6N. We selected 10 clinical samples to perform the miniPDX assay. Tumour tissues from HCC patients were separated and cultured, and eventually, these tumour cells were plated into mice to establish a miniPDX model. The relevant clinicopathological data of the miniPDX models are shown in Table S1. We divided the miniPDX models into five groups: vehicle D0, vehicle D7, sorafenib, RR‐170 and RR‐171. As shown in Figure S4, we detected the RLU (relative light unit) of each group. Based on these data, we further analysed the tumour/control (T/C) rate of each group. Compared with the vehicle control group, sorafenib and RR‐171 exhibited a significant inhibitory effect on HCC cells, while no significant changes were observed in the RR‐170 group (Figure 6O). The measurement of body weight from miniPDX models is provided in Figure S5. This evidence further indicated that RR‐171 had no obvious systemic toxicity in vivo, which was consistent with the data from transplanted tumour models experiment. Overall, these evidence indicated that RR‐171 significantly inhibited cell proliferation in vivo, which was consistent with the above results.

### 3.7 RR‐171 interacted with α‐tubulin and inhibited tubulin polymerization

**FIGURE 7:**
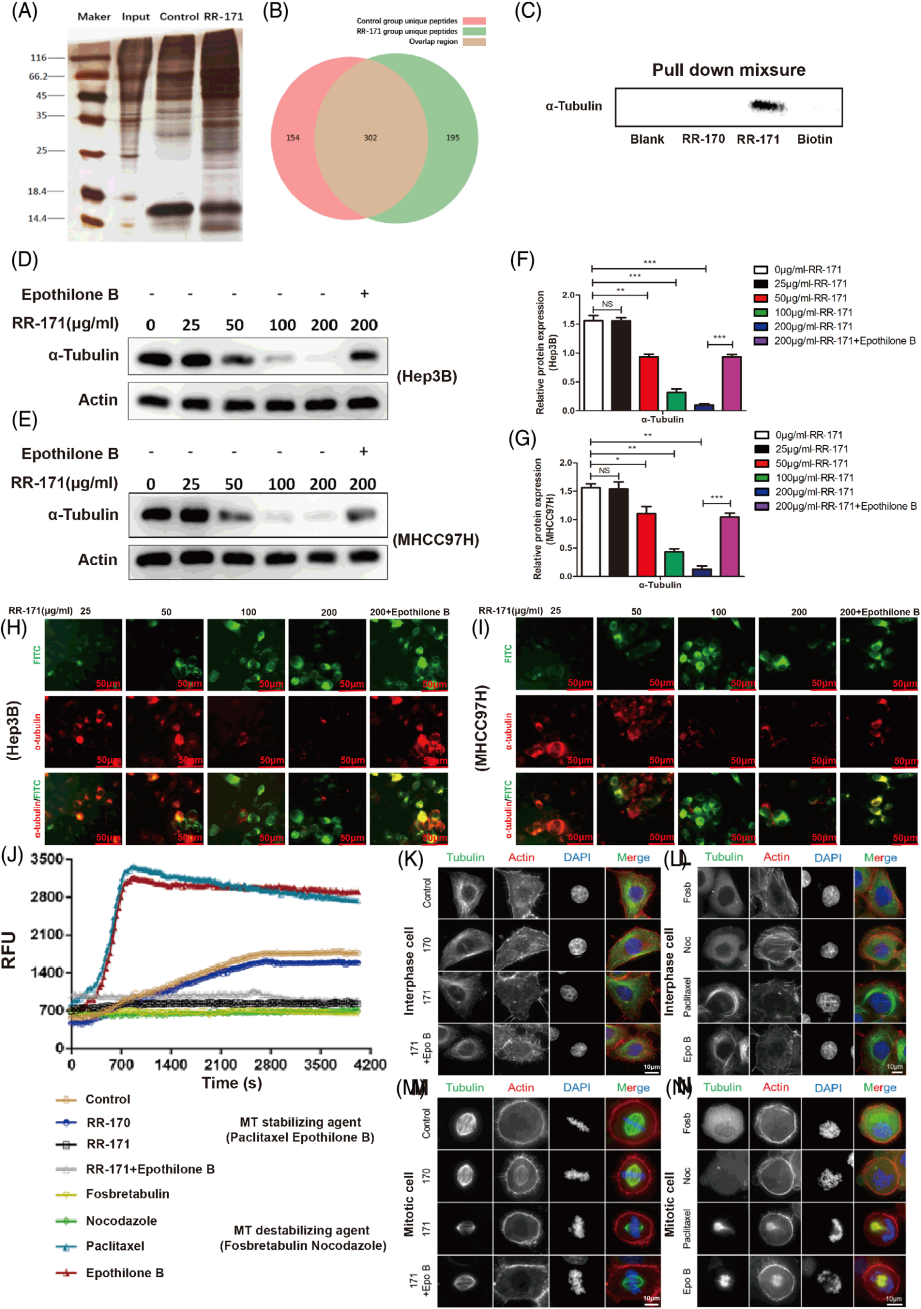
RR‐171 interacted with α‐tubulin and inhibited tubulin polymerization. (A) Protein bands were observed from different groups by silver staining detection after the pulldown assay. (B) Wayne diagram showing the differential proteins among the experimental samples. (C) Western blotting assay was used to detect α‐tubulin in pulldown mixtures of different groups. (D,E) Analysis of Western blotting showing α‐tubulin expression in HCC cells treated with different concentrations of RR‐171 and the microtubule stabilizer Epothilone B. (F,G) Quantification of D and E from the Western blotting experiments. The data are presented as the mean ± SEM. (H,I) Cell immunofluorescence assays were used to observe the colocalization of RR‐171 and α‐tubulin in different groups. Scale bar: 50 μm. (J) A tubulin polymerization assay kit was used to measure the RFU (relative fluorescence units) values of Hep3B cells, which were treated with RR‐170, RR‐171, RR‐171 + epothilone B, fosbretabulin, nocodazole, paclitaxel and epothilone B. (K–N) Analysis of cell immunofluorescence assays observing the aggregation of microtubules during the interphase and mitotic phases in different groups. Scale bar: 10 μm. *p < 0.05, **p < 0.01

To explore the mechanism by which RR‐171 induces tumour cell death, pulldown experiments were conducted to identify the potential targeting proteins of RR‐171. After silver staining detection, we found that there were many different bands in the experimental group compared with other groups, and these bands could probably contain the proteins binding with RR‐171 (Figure 7A). Figure 7B shows the Wayne diagram of the differential proteins among the experimental samples. According to the results, a total of 651 proteins were identified, among which 302 proteins were identified in both groups at the same time. The numbers of unique proteins identified in the RR‐171 group and the control group were 195 and 154, respectively. After mass spectrometry detection and scoring, α‐tubulin was selected as the subject of further study (Table 2). Representative secondary spectrograms of the α‐tubulin protein are detailed in Figure S6, which proves that α‐tubulin was identified by mass spectrometry. After verification by Western blotting assay, α‐tubulin was successfully detected in pull‐down mixtures of the RR‐171 group, but α‐tubulin was not detected in the other groups (Figure 7C).

**TABLE 2:** Analysis of mass spectrometry results showing the top five potential targets that scored high

Gene name	Score	Length	Coverage (%)	Peptide
UGGG1	36.02	1555	17.3	18
NUCB1	35.86	461	44.25	20
TPM2	34.18	284	41.9	16
TBA1A	28.80	451	44.57	15
PCYOX	22.10	505	27.72	11

To verify our hypothesis, we tested α‐tubulin expression in cell lysates treated with RR‐171 by Western blotting. Unexpectedly, we found that α‐tubulin expression decreased gradually with increasing RR‐171 in cell lysates. However, the situation was completely reversed by the use of the microtubule stabilizer epothilone B (Figure 7D–G). To determine the interaction between RR‐171 and α‐tubulin more intuitively, we used an immunofluorescence assay to clarify the colocalization relationship between them in cells. As shown in Figure 7H,I, the results were consistent with the results of the Western blotting assay. These data proved that RR‐171 could interact with α‐tubulin in HCC cells.

To determine the direct effects of RR‐171 on tubulin polymerization, we used a tubulin polymerization assay kit to detect whether RR‐171 affects microtubule dynamics in HCC cells. As shown in Figure 7J, polymerized tubulin was increased in the MT stabilizing agent groups (paclitaxel and epothilone B) compared with the control group and RR‐170 group. Conversely, polymerized tubulin was decreased in the MT destabilizing agent groups and RR‐171 group compared with the control group. Epothilone B reversed the effects of RR‐171 on tubulin polymerization. To visually test the effects of RR‐171 on tubulin polymerization, we observed microtubule aggregation during the interphase and mitotic phases using an immunofluorescence assay. The results indicated that RR‐171 significantly inhibited microtubule aggregation during the mitotic phase, but the inhibitory effect of RR‐171 on tubulin was not evident in interphase. Moreover, RR‐171 did not show a remarkable effect on actin in either the interphase or mitotic phase (Figure 7K–N). These data suggested that RR‐171 could interact with α‐tubulin and inhibit tubulin polymerization in HCC cells.

### 3.8 RR‐171 activated the NF‐Kappa B pathway in HCC cells

**FIGURE 8:**
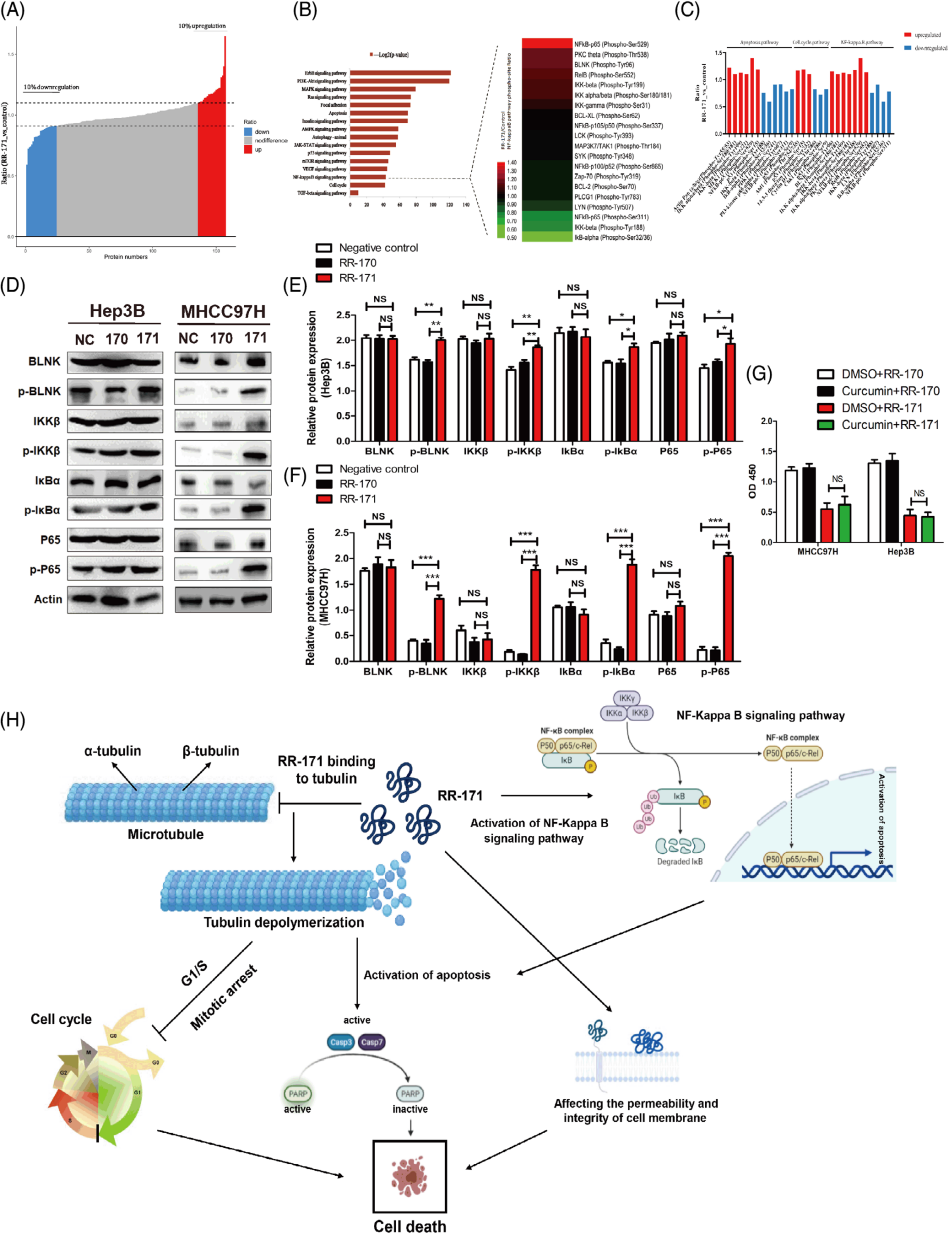
RR‐171 activated the NF‐Kappa B signalling pathway in HCC cells. (A) Analysis of a phosphospecific protein microarray showing more than 10% of these proteins whose phosphorylation levels were upregulated or downregulated in HCC cells treated with RR‐171, which were labelled red and blue, respectively. (B) KEGG analysis of proteins in the phosphospecific protein microarray showing cancer‐related signalling pathways. A heatmap was formed according to the ratio values of these phosphorylated proteins in the NF‐kappa B signalling pathway. The red block represents a high ratio, and the green block represents a low ratio in the heatmap. (C) Analysis of a phosphospecific protein microarray showing the changes in several key phosphoproteins crucial for the NF‐kappa B pathway, cell cycle pathway and apoptosis pathway. (D) Analysis of Western blotting showing the phosphorylation levels of these proteins (BLNK, IKKβ, IκBα and P65), which play a key role in the NF‐kappa B signalling pathway. (E,F) Quantification of D from the Western blotting experiments. The data are presented as the mean ± SEM. (G) CCK‐8 assay showing the OD_450_ of HCC cell lines treated with curcumin in four groups (DMSO + RR‐170 group, Curcumin + RR‐170 group, DMSO + RR‐171 group and Curcumin + RR‐171 group). (H) Proposed model showing the general process by which RR‐171 induces HCC cell death. *p < 0.05, **p < 0.01, ***p < 0.001

To further uncover the underlying signalling pathway by which RR‐171 induces tumour cell death, a phosphospecific protein microarray was used. As shown in Figure 8A, we observed more than 10% of these proteins whose phosphorylation levels were upregulated or downregulated in HCC cells treated with RR‐171. We focused on potential cancer‐related pathways through microarray analysis. Through gene ontology (GO) and Kyoto Encyclopedia of Genes and Genomes (KEGG) analyses, we focused on the NF‐kappa B pathway, which is critical to cell cycle progression and cell apoptosis in cancer cells. The results indicated that the NF‐Kappa B pathway was activated by RR‐171 in HCC cells (Figure 8B). Except for the NF‐kappa B signalling pathway, these proteins in the phosphorylated form were involved in the cell cycle pathway and apoptosis pathway, and many phosphorylated proteins overlapped in the intersection of these pathways (Figure 8C). To demonstrate our hypothesis, a Western blotting assay was conducted to test the phosphorylation status and levels of the indicated proteins, which are crucial to regulate the NF‐kappa B signalling pathway. The results indicated that the NF‐Kappa B signalling pathway was activated by RR‐171 in HCC cells (Figure 8D–F). Furthermore, we wondered whether RR‐171 could cause cell death after blocking the NF‐Kappa B signalling pathway. So we used curcumin to block the NF‐Kappa B signalling pathway, after which RR‐171 still inhibit tumour growth significantly (Figure 8G). These evidence indicated that RR‐171 leading to tumour cell death is a complicated process, which could be involved in multiple biological activities.

## 4 DISCUSSION

Hepatocellular carcinoma (HCC) is one of the most common malignant tumours and major causes of cancer‐related death worldwide, and there is an urgent need for new drugs with high efficiency, high selectivity and low toxicity in clinical practice. At present, many drugs for the treatment of cancer are ineffective, and many cancers can develop drug resistance, leading to increased side effects. The efficacy, stability and low toxicity of natural peptides or modified peptides make them a new choice for clinical application.^19^ Although it is believed that antitumour peptides are effective against tumour cells without damaging normal physiological functions, the development of selectivity has always been a challenge.^20^

There has been little research on the relationship between umbilical cord blood and liver‐related diseases, let alone liver cancer. Human umbilical cord serum contains higher concentrations of multiple cytokines and growth factors than peripheral blood serum, and these cytokines and growth factors have anti‐inflammatory and antiapoptotic effects.^21^ Exosomes derived from umbilical cord blood stem cells have been reported to ameliorate IL‐6‐mediated acute liver injury.^22^ Human umbilical cord serum can reduce gentamicin‐induced hepatotoxicity by restoring peripheral oxidative damage and the inflammatory response in rats.^23^ Accidentally, we found that umbilical cord serum has a certain inhibitory effect on HCC cells. To further analyse human umbilical cord serum and verify its effect on tumour biology, we collected and measured the bioactivity of human umbilical cord serum by proteomics biotechnology. Eventually, a primitive peptide with certain biological activity was screened out. On this basis, we enhanced the solubility of the original peptide and obtained the modified peptide RR‐171.

To further exclude nonspecific factor effects such as concentration and acid base, we designed a control sequence based on RR‐171. The control peptide RR‐170 was obtained by replacing valine and leucine with lysine. The physicochemical properties of RR‐171 and RR‐170 were analysed by the online software INNOVAGEN, and the secondary structure of the peptides was predicted by circular dichroism spectroscopy. The physicochemical properties could be applied in the development of molecularly targeted drugs according to the composition of amino acid residues, molecular weight, isoelectric point, net charge and secondary structure of peptides.^24^ Then, RR‐171 was evaluated for the growth inhibition test by the CCK‐8 assay. We found that RR‐171 can broadly inhibit the growth of tumour cells, among which HCC cells were most sensitive to RR‐171. Meanwhile, the toxicity of RR‐171 to human normal liver cells was much lower than the toxicity of RR‐171 to liver cancer cells. Therefore, HCC was chosen as the research object of this study. We found that the cytotoxicity of RR‐171 had concentration‐dependent and time‐dependent characteristics, and RR‐171 showed significant growth inhibition of HCC cells after 12 h and reached a peak at 48 h through different kinds of experimental verification in vitro.

Then, we used a flow cytometry assay to evaluate whether RR‐171 played a regulatory role in the process of HCC cell cycle progression and apoptosis. The evidence indicated that RR‐171 inhibited the G1/S phase transition and induced apoptosis of HCC cells. Subsequently, we found that RR‐171 could activate related factors of the cell cycle and apoptosis through Western blotting detection, which also confirmed the above results. Apoptosis is a type of programmed cell death, and detailed study of apoptosis‐related elements and signals will help provide in‐depth knowledge for the further development of cancer‐related drugs. However, cell death is not only caused by apoptosis; necrosis and oncosis are also two important forms of cell death. To uncover the specific death mechanism caused by RR‐171, necrosis inhibitors, oncosis inhibitors and apoptosis inhibitors were used to coculture HCC cells treated with RR‐171. This evidence proved that RR‐171 induced cell death through apoptosis. Numerous anticancer peptides induce apoptosis of cancer cells or promote rupture of the cell membrane, which eventually leads to cell death.^25^, ^26^, ^27^ The cancer cell membrane presents stronger membrane fluidity on the cell membrane than normal cells, and the surface of cancer cells is more abundant with microvilli, increasing the cell surface area.^28^, ^29^ The surface of cancer cells is negatively charged, while the surface of normal cells is electrically neutral, which makes the membrane of cancer cells more unstable when they come in contact with anticancer peptides, making the cell membrane more prone to rupture.^30^, ^31^, ^32^ LDH was released when the cell membrane was destroyed, and an LDH leakage assay was conducted to identify the integrity of the cell membrane. After treatment with RR‐171, the LDH level of HCC cells continued to increase and reached a peak at 48 h. Moreover, the micropores on the cellular surface increased, as observed by SEM. Thus far, RR‐171 not only induces apoptosis in HCC cells but also has a cell‐penetrating capacity that could be one of the causes of cancer cell death.

RR‐171 exhibited fair antitumour activity in vitro, so we were curious about its biological activity in vivo. First, we explored the tumour‐targeting ability and biodistribution of RR‐171 in vivo. The results showed that the fluorescence intensities of the liver and tumour tissue were the strongest, indicating that RR‐171 could significantly aggregate in solid tumours. The superior tumour‐targeting ability makes RR‐171 a promising agent for clinical application; for instance, RR‐171 could be applied for the location of cancer in the human body. Compared with traditional chemotherapeutic drugs, anticancer peptides have an obvious advantage in that they are less toxic, which makes them more popular in clinical applications. We found that RR‐171 had no obvious systemic toxicity in vivo. RR‐171 also exhibited a remarkable tumour inhibitory effect not only in subcutaneous xenograft tumours of nude mice but also in the miniPDX assay in vivo. The PDX model has attracted increasing attention for cancer research.^33^ Currently, it is urgent to search for appropriate preclinical models in preclinical trials of new drug therapies. The PDX model can not only constitute a paraclinical model that retains the histological and genetic characteristics of the original patient but also maintains stability in the passage process.^34^

In terms of biological activity, RR‐171 exhibited impressive antitumour activity in vitro and in vivo. RR‐171 can also induce apoptosis in HCC cells, and its cell‐penetrating capacity may play a pivotal role in regulating cell death. The interaction between RR‐171 and the cell membrane was the premise of its anticancer activity, but the specific mechanism of RR‐171 leading to tumour cell death still needs to be explored. To identify the exact binding targets of RR‐171, a pull‐down assay was conducted. Through mass spectrometry detection and scoring, α‐tubulin was chosen as the potential target of RR‐171. Tubulin is a major component of microtubules, which participate in various cellular processes, including cell division, cell replication and intracellular material transport.^35^, ^36^ Tubulin‐binding compounds could affect cell cycle progression through the inhibition of the dynamic formation of mitosis, ultimately activating the downstream apoptotic signalling pathway.^37^ Moreover, microtubules are essential to maintain the stability and permeability of the cell membrane.^38^, ^39^ To determine whether RR‐171 affects microtubule dynamics, we tested the protein level of α‐tubulin in HCC cells treated with RR‐171. The loss of microtubules increased with increasing concentrations of RR‐171, while the situation was reversed with the use of a microtubule stabilizer, as observed by Western blotting and immunofluorescence. Then, we evaluated whether RR‐171 could disturb tubulin polymerization directly through a tubulin polymerization assay. We found that RR‐171 significantly inhibited microtubule aggregation during the mitotic phase, but the inhibitory effect was not obvious in interphase. We found that RR‐171 could activate the NF‐Kappa B signalling pathway through phosphor‐specific protein microarray analysis.

In conclusion, we designed a novel peptide RR‐171 derived from human umbilical cord serum, which exhibited significant antitumour activity against HCC in vivo and in vitro. RR‐171 could not only induce apoptosis and inhibit tubulin polymerization in HCC cells but also activate the NF‐kappa B signalling pathway in HCC cells. This evidence indicated that RR‐171 may have therapeutic potential for the treatment of HCC, which provides a new horizon for the clinical application of HCC.

## CONFLICT OF INTEREST

The authors declare that they have no competing interests.

## AUTHOR CONTRIBUTIONS

Donglie Zhu and Yong Chen designed the study. Donglie Zhu, Cheng Fang, Zelong Yang and Duoduo Liu performed the experiments. Donglie Zhu, Cheng Fang, Yanjie Ren and Biliang Chen contributed to data analysis. Donglie Zhu, Fengrui Yang, Xiangxia Miao and Shi Zheng performed data interpretation. Mingzuo Jiang, Yong Chen and Xuebiao Yao contributed to the critical revision of the manuscript. Donglie Zhu wrote the manuscript. All authors approved the final version of the manuscript.

## Supporting information


**FIGURE S1** Mass spectrometry analysis and HPLC chromatograms of the peptides. (A,B) Mass spectrometry analysis of RR‐170 and RR‐171. (C,D) HPLC chromatograms of purified RR‐170 and RR‐171. Concentration, conversion. The purity of the peptides used in the experiments was over 98%

**FIGURE S2** Flow chart for phosphorspecific protein microarray analysis. Proteins were extracted from HCC cells treated with RR‐171, and cells treated with complete medium were used as a negative control. Then, the protein samples were labelled with biotin followed by hybridization to the microarray. Scanning of the chips was performed using the Agilent Microarray Scanner (Agilent Technologies, Santa Clara, CA, USA)

**FIGURE S3** Detection of the viability of cancer cell lines treated with RR‐171 or RR‐170. HepG2, liver cancer cell line; Huh7, liver cancer cell line; HCCLM3, liver cancer cell line; HCCLM6, liver cancer cell line; PANC‐1, pancreatic cancer cell line; SW480, colon cancer cell line; A2780, ovarian cancer cell line; Skbr‐3, breast cancer cell line; A549, lung cancer cell line; MKN45, gastric cancer cell line. The data are presented as the mean ± SEM. *p < 0.05, **p < 0.01, ***p < 0.001

**FIGURE S4** RLU (relative light unit) of miniPDX models (*n* = 10) from different groups

**FIGURE S5** Body weight of miniPDX models (*n* = 10) from different groups

**FIGURE S6** Representative secondary spectrograms of α‐tubulin

**TABLE S1** Relevant clinicopathological data of MiniPDX models

## Data Availability

The data used to support the fundings of this study are available from the corresponding author upon request.
