# Supplementary figures and images for: Tubulin‐binding peptide RR‐171 derived from human umbilical cord serum displays antitumor activity against hepatocellular carcinoma via inducing apoptosis and activating the NF‐kappa B pathway
Source: Cell Prolif. 2022 May 3;55(5):e13241. doi: 10.1111/cpr.13241 (PMC9136518; doi:10.1111/cpr.13241)

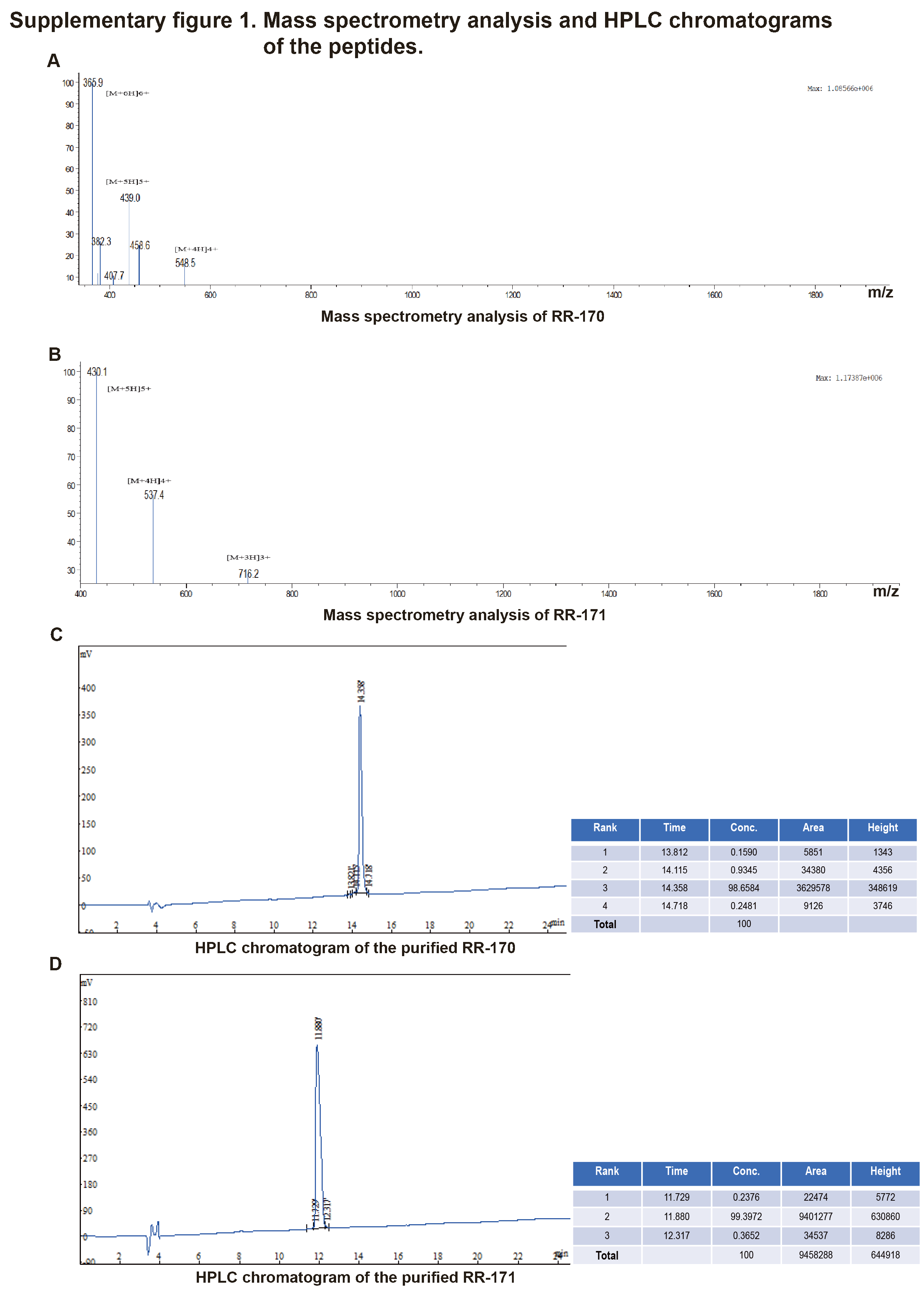

Supplement: Supplementary file 1 — FIGURE S1 Mass spectrometry analysis and HPLC chromatograms of the peptides. (A,B) Mass spectrometry analysis of RR‐170 and RR‐171. (C,D) HPLC chromatograms of purified RR‐170 and RR‐171. Concentration, conversion. The purity of the peptides used in the experiments was over 98% [file CPR-55-e13241-s007.gif]

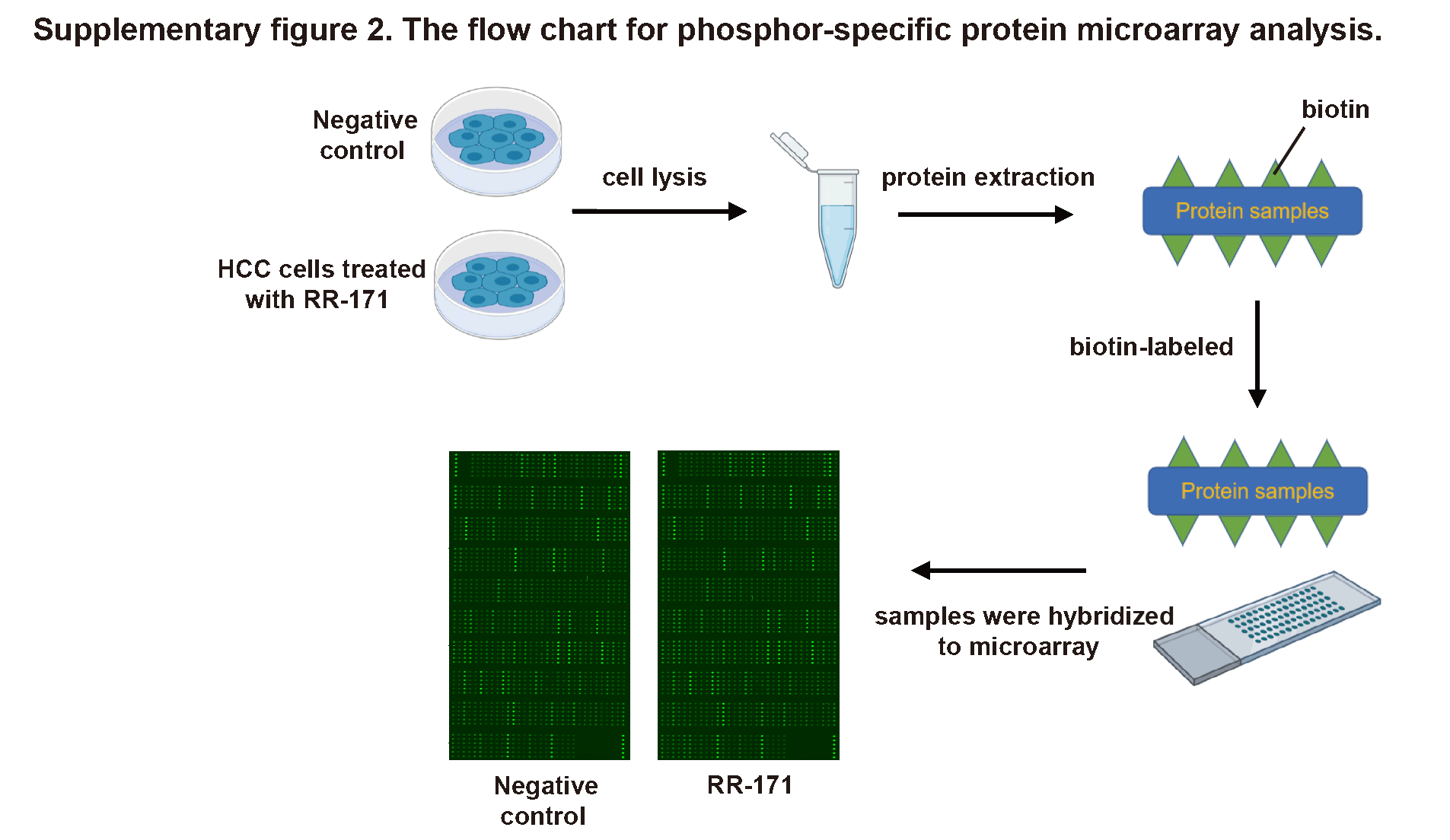

Supplement: Supplementary file 2 — FIGURE S2 Flow chart for phosphorspecific protein microarray analysis. Proteins were extracted from HCC cells treated with RR‐171, and cells treated with complete medium were used as a negative control. Then, the protein samples were labelled with biotin followed by hybridization to the microarray. Scanning of the chips was performed using the Agilent Microarray Scanner (Agilent Technologies, Santa Clara, CA, USA) [file CPR-55-e13241-s001.gif]

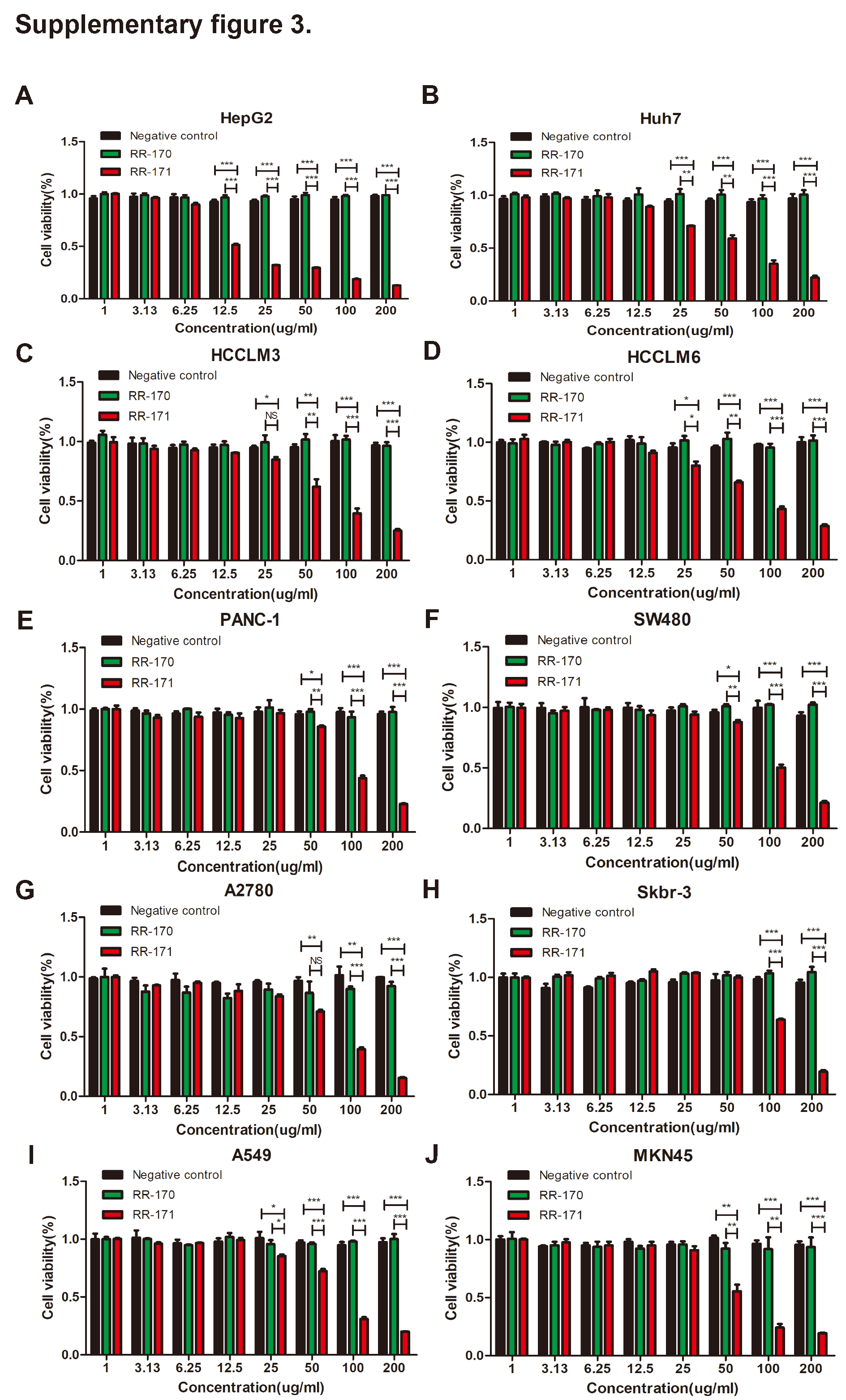

Supplement: Supplementary file 3 — FIGURE S3 Detection of the viability of cancer cell lines treated with RR‐171 or RR‐170. HepG2, liver cancer cell line; Huh7, liver cancer cell line; HCCLM3, liver cancer cell line; HCCLM6, liver cancer cell line; PANC‐1, pancreatic cancer cell line; SW480, colon cancer cell line; A2780, ovarian cancer cell line; Skbr‐3, breast cancer cell line; A549, lung cancer cell line; MKN45, gastric cancer cell line. The data are presented as the mean ± SEM. *p < 0.05, **p < 0.01, ***p < 0.001 [file CPR-55-e13241-s003.gif]

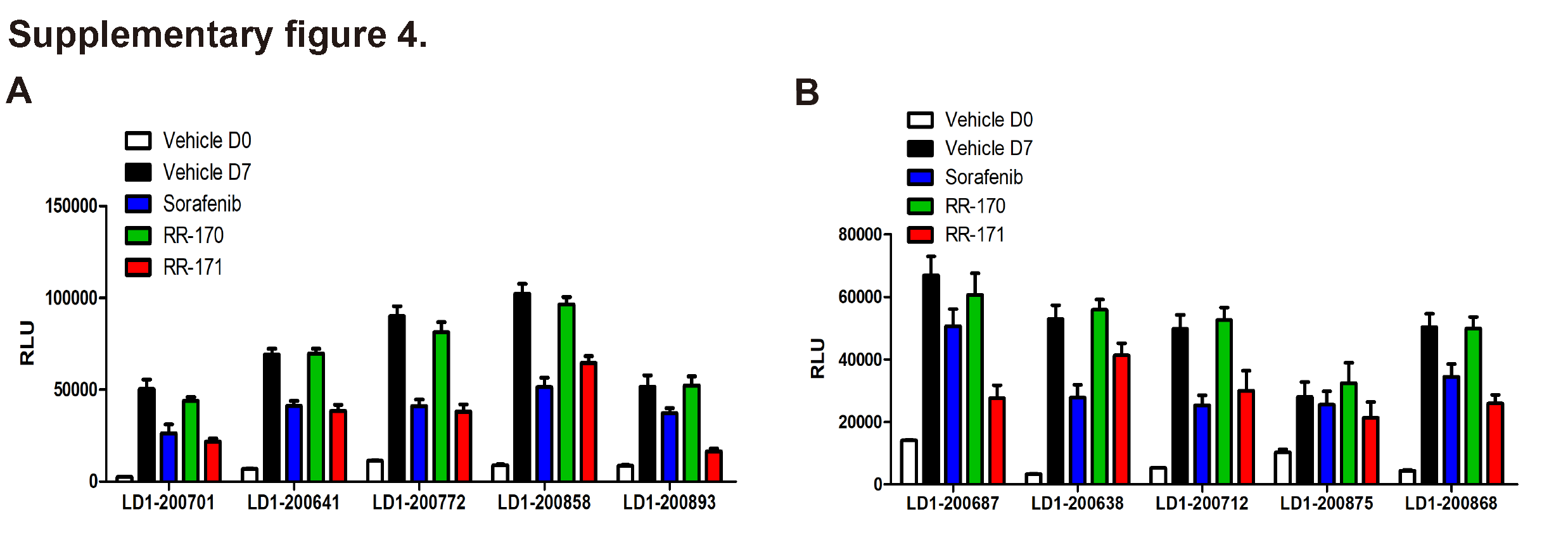

Supplement: Supplementary file 4 — FIGURE S4 RLU (relative light unit) of miniPDX models (n = 10) from different groups [file CPR-55-e13241-s002.gif]

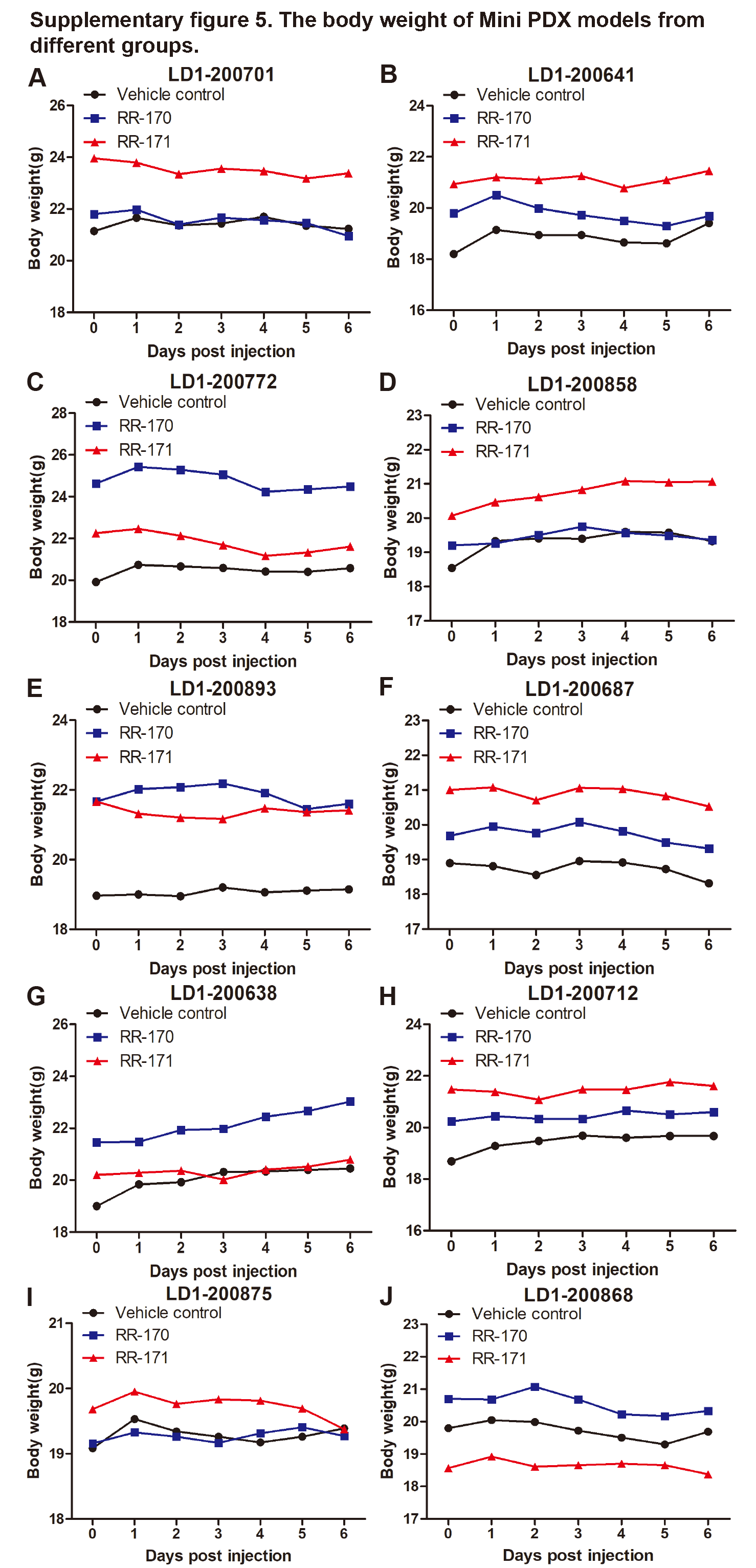

Supplement: Supplementary file 5 — FIGURE S5 Body weight of miniPDX models (n = 10) from different groups [file CPR-55-e13241-s006.gif]

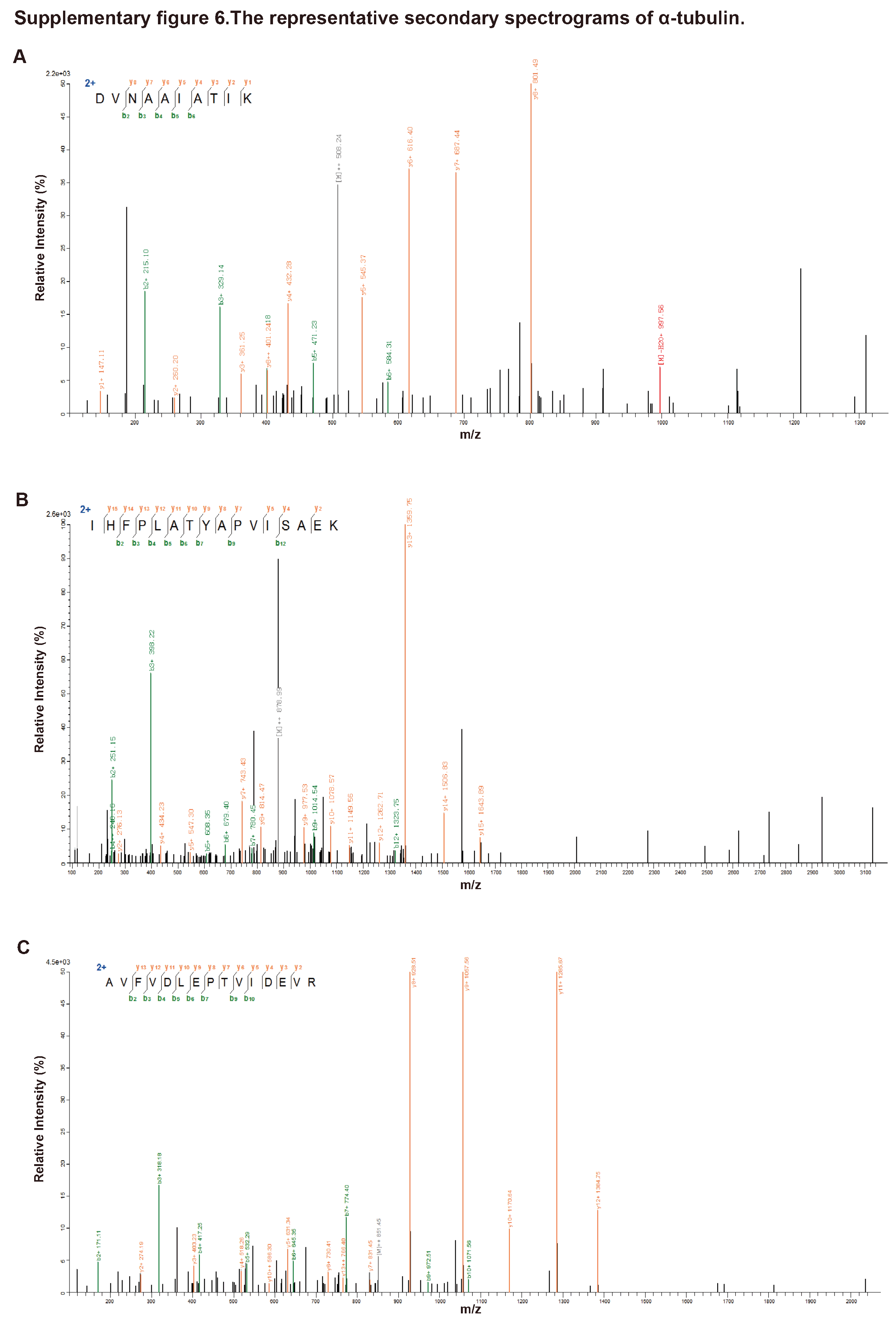

Supplement: Supplementary file 6 — FIGURE S6 Representative secondary spectrograms of α‐tubulin [file CPR-55-e13241-s005.gif]
