# Supplementary material for: Tubulin‐binding peptide RR‐171 derived from human umbilical cord serum displays antitumor activity against hepatocellular carcinoma via inducing apoptosis and activating the NF‐kappa B pathway
Source: Cell Prolif. 2022 May 3;55(5):e13241. doi: 10.1111/cpr.13241 (PMC9136518; doi:10.1111/cpr.13241)
Supplement: Supplementary file 7 — TABLE S1 Relevant clinicopathological data of MiniPDX models [file CPR-55-e13241-s004.docx]

Supplementary table 1. The relevant clinicopathological data of Mini PDX models.

| Number | Sex | Age (year) | Clinical pathological classification |
| --- | --- | --- | --- |
| LD1-200701 | Male | 84 | Primary liver cancer (poorly differentiated) |
| LD1-200641 | Male | 57 | Primary liver cancer (moderately differentiated) |
| LD1-200772 | Female | 55 | Primary liver cancer (poorly differentiated) |
| LD1-200858 | Male | 69 | Primary liver cancer (poorly differentiated) |
| LD1-200893 | Male | 53 | Primary liver cancer (moderately differentiated) |
| LD1-200687 | Female | 34 | Primary liver cancer (poorly differentiated) |
| LD1-200638 | Female | 55 | Primary liver cancer (poorly differentiated) |
| LD1-200712 | Male | 40 | Primary liver cancer (poorly differentiated) |
| LD1-200875 | Male | 45 | Primary liver cancer (poorly differentiated) |
| LD1-200868 | Male | 61 | Primary liver cancer (moderately differentiated) |
